# The Utility of Strain Echocardiography in the Diagnosis of Pediatric Myocarditis: A Systematic Review and Meta‐Analysis

**DOI:** 10.1111/echo.70443

**Published:** 2026-04-15

**Authors:** Krzysztof Macierzanka, Abhisekh Chatterjee, Ahmer Adnan, Hassan Marzook, Rafay Yousaf, Craig Laurence, Javier Gavela

**Affiliations:** ^1^ Faculty of Medicine Imperial College London London UK; ^2^ National Heart and Lung Institute Imperial College London London UK; ^3^ Department of Surgery and Cancer Imperial College London London UK; ^4^ Department of Cardiology Great Ormond Street Hospital London UK; ^5^ Department of Cardiac Intensive Care Great Ormond Street Hospital London UK

**Keywords:** diagnosis, echocardiography, myocarditis, speckle tracking, strain

## Abstract

**Purpose:**

The clinical presentation of pediatric myocarditis is heterogeneous, and diagnosis can be elusive. Diagnostic criteria feature cardiac magnetic resonance (CMR) as the imaging gold‐standard, but this is not always feasible. Strain echocardiography has been shown to aid in the diagnosis of suspected myocarditis. This report aimed to synthesize available literature regarding the use of strain echocardiography for the diagnosis of pediatric myocarditis, and to systematically review the concordance between strain echocardiography and CMR (PROSPERO CRD42024596691).

**Methods:**

A systematic search of MEDLINE, Embase, and Scopus was conducted up to July 2025 to identify studies describing strain echocardiography for diagnosis of pediatric myocarditis, and thereby estimate mean differences (MDs) in conventional and strain echocardiographic values between pediatric myocarditis and controls.

**Results:**

Seven studies were included (three case–control [myocarditis *n* = 119, control *n* = 50] and four cohort [*n* = 101]). The MD in left ventricular ejection fraction (LVEF) and global longitudinal strain (LV‐GLS) between pediatric myocarditis and controls was −4.3% (−10.5% to 1.9%) and −5.2% (−7.7% to −2.8%), respectively. The mean pooled LVEF and LV‐GLS for the pediatric myocarditis population was 61.9% (58.1% to 65.6%) and −17.0% (−19.3% to −14.8%), respectively. Subgroup analysis on LVEF status (normal vs. abnormal) showed differences in mean LVEF (*p* = 0.042), but not in the mean LV‐GLS (*p* = 0.583). Throughout reports which included CMR imaging, strain echocardiography reliably and consistently identified CMR‐positive myocardial injury.

**Conclusion:**

Strain echocardiography is a valuable tool for the assessment of suspected pediatric myocarditis, showing good concordance with CMR and identifying myocardial dysfunction before conventional echocardiographic measures decline.

## Graphical abstract

Strain echocardiographic assessment yields reduced LV‐GLS values across the pediatric myocarditis population. These remain abnormal irrespective of LVEF status. There is good concordance between strain echocardiography and CMR. Strain echocardiography is a useful diagnostic tool in suspected pediatric myocarditis. CMR, cardiac magnetic resonance; LVEF, left ventricular ejection fraction; LV‐GLS, left ventricular global longitudinal strain.

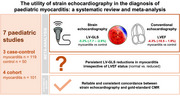

**Abbreviations**
CMR: cardiac magnetic resonance
ECG: electrocardiogram
LVEDD: left ventricular end‐diastolic diameter
LVEF: left ventricular ejection fraction
LV‐GCS: left ventricular global circumferential strain
LV‐GLS: left ventricular global longitudinal strain
MIS‐C: multisystem inflammatory syndrome in children
PIMS: pediatric inflammatory multisystem syndrome
PRISMA: Preferred Reporting Items for Systematic Reviews and Meta‐Analyses
QUADAS‐2: Quality Assessment of Diagnostic Accuracy Studies 2
STE: speckle‐tracking echocardiography
TTE: transthoracic echocardiography

## 1 Introduction

Myocarditis is an inflammatory disease of the myocardium, most commonly with viral or idiopathic etiology. It is a significant and often under‐recognized cause of acute and chronic cardiac dysfunction in children [1]. The clinical presentation of myocarditis in the pediatric population is heterogeneous and variable, and encompasses non‐specific symptoms such as fatigue, failure to thrive, increased work of breathing, or syncope [2]. Therefore, it can be easily missed in early or subclinical stages. Late diagnosis can prevent timely initiation of supportive measures in the acute setting. This can lead to adverse remodeling and dilated cardiomyopathy, which remains a leading indication for pediatric heart transplantation [3].

Diagnosis is therefore crucial to prevent progression and guide timely management. The most common diagnostic algorithms often rely on a combination of clinical suspicion, elevated biomarkers such as troponin, electrocardiogram (ECG) findings, and advanced imaging [4], but are compounded by the limitations of these methods: ECG changes and troponin elevations may be absent or transient [5], and conventional echocardiographic indices such as left ventricular ejection fraction (LVEF) can remain normal until late in the disease process [6, 7].

Cardiac magnetic resonance (CMR) has emerged as the non‐invasive gold‐standard diagnostic approach [4], focusing on the detection of myocardial edema and fibrosis [8, 9]. However, the practical utility of CMR in the acute pediatric setting may be constrained by resource limitation, patient stability and logistical factors [10, 11].

Hence, transthoracic echocardiography (TTE) remains the imaging modality of choice for acute pediatric myocarditis due to its accessibility, portability, and lack of radiation exposure. Yet, its conventional indices provide only global volumetric measures of cardiac function. For example, LVEF predominantly reflects radial thickening of the ventricular wall and may not detect early regional or layer‐specific dysfunction [12], and hence may be insensitive in non‐fulminant myocarditis cases [13]. This has led to the development of advanced techniques, such as speckle‐tracking echocardiography (STE), which provides a quantitative assessment of myocardial deformation (strain) [14]. STE has already demonstrated diagnostic and prognostic value in several pediatric cardiac conditions, including early detection of Kawasaki disease [15], cardiomyopathy in Duchenne muscular dystrophy [16], and anthracycline‐related cardiotoxicity [17]. These applications highlight its diagnostic sensitivity for subclinical myocardial injury before traditional parameters suffer.

Recent work in adults has demonstrated that strain parameters, particularly global longitudinal strain (GLS), differentiate patients with myocarditis from healthy control subjects [18]. However, whether similar diagnostic accuracy exists in the pediatric population remains uncertain. Therefore, we conducted this systematic review and meta‐analysis to 1) determine the ability of STE to differentiate pediatric myocarditis from healthy controls, and 2) assess how closely echocardiographic strain corresponds with CMR to evaluate its potential as a non‐invasive surrogate when CMR is unfeasible.

## 2 Methods

### 2.1 Protocol and Search Strategy

This review was conducted in accordance with the Meta‐analysis of Observational Studies in Epidemiology guidelines [19] and the Preferred Reporting Items for Systematic Reviews and Meta‐Analyses (PRISMA) statement (Table S1) [20]. The protocol for this study was registered a priori on the International Prospective Register of Systematic Reviews (PROSPERO, CRD42024596691).

A systematic search of MEDLINE, Embase, and Scopus was conducted from database inception to July 2025 for studies reporting the use of strain echocardiography or STE in pediatric myocarditis. We used a combination of MeSH terms and keywords specific to our research question, including but not limited to “strain”, “gls”, “echocardiography”, “speckle‐tracking”, and “myocarditis”. No language filters were applied, and the gray literature was not searched. The full search strategy is outlined in the Supplementary Appendix.

### 2.2 Study Eligibility

#### 2.2.1 Inclusion Criteria

Only primary studies published in peer‐reviewed English‐language papers were included. Eligible studies included case–control, cohort, or case‐series designs (*n* ≥ 5) that enrolled pediatric patients (aged ≤18 years) with viral, idiopathic, or vaccine‐associated myocarditis and reported TTE data at or near the time of diagnosis to assess the viability of STE as a diagnostic tool in pediatric myocarditis. Studies were included regardless of whether a healthy control population was present.

#### 2.2.2 Exclusion Criteria

Studies pertaining exclusively to multisystem inflammatory syndrome in children/pediatric inflammatory multisystem syndrome (MIS‐C/PIMS) or Kawasaki disease‐related myocarditis were excluded based on their heterogeneous clinical course and recovery times [21, 22]. We also excluded all other types of report not mentioned above, as well as those only in abstract form.

### 2.3 Screening and Extraction

All retrieved records were imported into the Covidence software [23] for reference management and screening. Following deduplication of references, initial title and abstract screening, as well as subsequent full‐text screening, was undertaken by two reviewers for each study (K.M. and A.C.) who were blinded to each other's decisions. Discrepancies were resolved by discussion and consensus.

Data extraction was performed independently by four investigators (A.C., A.A., H.M., and R.Y.), with each article extracted twice. Extracted data included study characteristics, baseline demographic characteristics, echocardiographic parameters, and available baseline CMR parameters. Discrepancies were resolved by K.M. When studies included mixed patient populations, only data from myocarditis or relevant subgroups were retained (see Supplementary Appendix for details). All extracted TTE indices were converted to absolute values before meta‐analysis to ensure consistency of directionality. Study authors were not contacted for missing or subgroup data.

### 2.4 Outcomes

The primary outcome was the ability of echocardiographic indices to discern pediatric myocarditis from healthy controls, expressed as pooled mean differences (MDs). Secondary outcomes included establishing pooled reference values for echocardiographic indices among pediatric myocarditis patients, as well as a systematic narrative synthesis of the relationship between echocardiographic and CMR findings in this population.

### 2.5 Study Quality Appraisal

Study quality was independently assessed by three investigators (A.A., H.M., and R.Y.) using the Quality Assessment of Diagnostic Accuracy Studies 2 (QUADAS‐2) risk of bias tool [24]. Risk of bias was evaluated across four domains (patient selection, index test, reference standard, and flow and timing).

### 2.6 Statistics

The ability of TTE indices to differentiate pediatric myocarditis from healthy control patients was assessed through MD meta‐analysis inclusive of all case–control studies only. The pediatric myocarditis references were ascertained through pooling untransformed study‐level means from the myocarditis arms of all studies, irrespective of design. Random‐effects models (DerSimonian–Laird method) were used throughout. Pooled estimates are presented with 95% confidence intervals (CIs).

Statistical heterogeneity was evaluated using Cochran's *Q* statistic, *τ* ^2^, and the *I* ^2^ statistic. Sources of heterogeneity were analyzed using exploratory subgroup analysis, and linear and non‐linear meta‐regression (where applicable) examining the effects of mean patient age, subclinical presentation (normal LVEF only vs. all ranges of LVEF), and myocarditis etiology (viral/idiopathic vs. vaccine‐associated). Sensitivity analyses were performed to assess the impact of individual studies and study quality.

For studies reporting medians and interquartile ranges, means and standard deviations were imputed using established statistical methods [25].

All analyses were carried out in the R statistical environment (R Foundation for Statistical Computing) using the meta, splines, estmeansd, and tidyverse packages.

## 3 Results

### 3.1 Search Results

**FIGURE 1:**
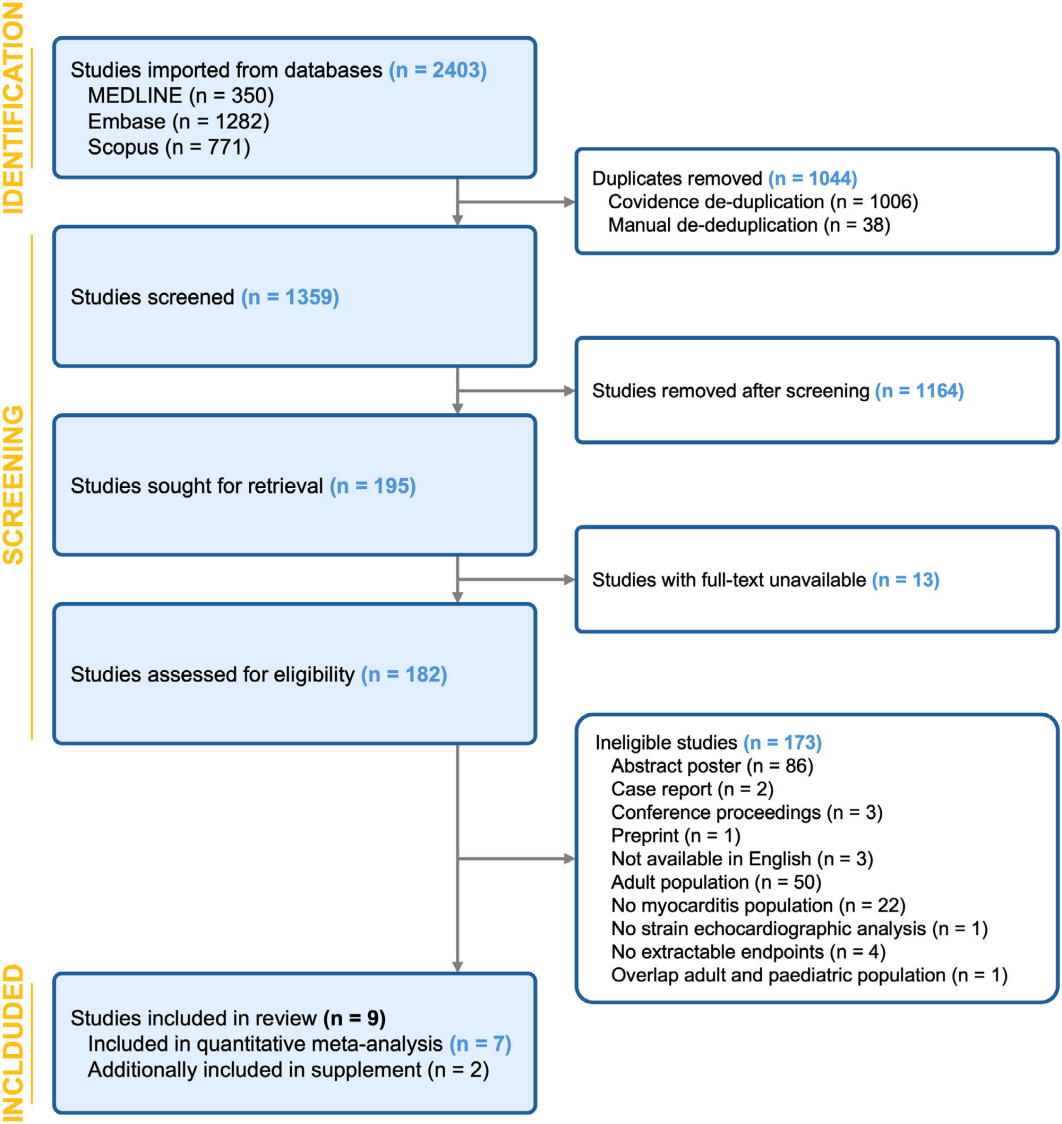
PRISMA flowchart. PRISMA, Preferred Reporting Items for Systematic Reviews and Meta‐Analyses.

The systematic search identified 2403 citations. After the removal of duplicates and screening of titles and abstracts, 195 studies underwent full‐text screening (Figure 1). Of these, nine met the inclusion criteria and were included in the present report (Table 1) [26, 27, 28, 29, 30, 31, 32, 33, 34]. Two studies [33, 34] were excluded from the quantitative meta‐analysis due to substantially younger cohorts and non‐comparable echocardiographic reference ranges [35]. Quantitative syntheses inclusive of these studies are presented in the Supplementary Appendix. Therefore, seven studies (three case–control [myocarditis *n* = 119, control *n* = 50] and four cohort studies [*n* = 101]) contributed to the main pooled analyses. Outside of these nine reports, a case–control study by Khoo et al. [36] was not included as it presented combined adolescent and adult TTE data that could not be separated for pediatric analysis.

**TABLE 1:** Included studies.

Report	Study design	Subjects (*n*)	Myocarditis definition	Age (years)
Uppu et al. 2015 [26]	Case–control	Myocarditis: 10 Control: 19	CMR confirmation **Only enrolled subjects with normal LVEF**	Myocarditis: 17.5, 14–18.6 Control: 14.5, 8.3–22.0 (median, range)
Gursu et al. 2019 [27]	Case–control	Myocarditis: 21 Control: 12	ESC Working Group on Myocardial and Pericardial Diseases criteria [37]	Myocarditis: 12.3 ± 3.3 Control: 11.4 ± 3.1 (mean ± SD)
Schauer et al. 2022 [28]	Case–control	Myocarditis: 88 Control: 19	Lake Louise 2009 [8]	Myocarditis: 15.6 ± 2.0 Control: 16.6 ± 2.6 (mean ± SD)
Chinali et al. 2020 [29]	Cohort	Myocarditis: 33	Lake Louise 2009 [8] **Only enrolled subjects with normal LVEF**	Myocarditis: 13, 10–16 (median, IQR)
Dionne et al. 2021 [30]	Cohort	Myocarditis: 15	“Chest pain and an elevated troponin level in the absence of an alternative diagnosis […] within 30 days of BNT162b2 messenger RNA COVID‐19 vaccine”	Myocarditis: 15, 12–18 (median, range)
Burešová et al. 2024 [31]	Cohort	Myocarditis: 20	Lake Louise 2018 [9]	Myocarditis: 14, 9–16.8 (median, IQR)
Alkan et al. 2025 [32]	Cohort	Myocarditis: 33	Not explicitly specified. Enrolled subjects “with a preliminary diagnosis of acute myocarditis” **Only enrolled subjects with normal LVEF**	Myocarditis: 14.7 ± 2.9 (mean ± SD)
*Bigg* et al. *2023* [33]	*Cohort*	*Myocarditis: 14*	*Abnormal LVEF and positive real‐time enteroviral PCR*	*Myocarditis: 9.5, 7–12 (days)* *(median, IQR)*
*Bhatia* et al. *2026* [34]	*Cohort*	*Myocarditis: 23*	*Acute myocarditis (ICD‐10 code B33.22)*	*Myocarditis: 3.8 ± 4.8* *(mean ± SD)*

*Note*: The two studies with substantially younger cohorts and non‐comparable echocardiographic reference ranges are highlighted.

Abbreviations: CMR, cardiac magnetic resonance; ESC, European Society of Cardiology; ICD, International Classification of Diseases; IQR, interquartile range; LVEF, left ventricular ejection fraction; PCR, polymerase chain reaction; SD, standard deviation.

### 3.2 Study Quality

Overall methodological quality was high. Based on the QUADAS‐2 assessment, all studies demonstrated low risk of bias across the majority of domains. The full assessment across domains for the included studies is available in the Supplementary Appendix (Table S2).

### 3.3 Myocarditis Definition and Study Characteristics

The principal characteristics of included studies are summarized in Table 1. Myocarditis definitions were heterogeneous but largely consistent with international diagnostic standards [8, 9]. All case–control studies, and most cohorts, employed CMR‐based definitions. Three studies [26, 29, 32] only recruited subjects with normal LVEF at presentation. One study [30] investigated vaccine‐associated myocarditis, whereas the remainder included viral or idiopathic cases. The variations in mean patient age, effect of varied global systolic function inclusion criteria, and etiology are explored in subsequent analyses.

### 3.4 Left Ventricular Systolic Indices

From the included studies, the only consistently reported left ventricular systolic indices available for meta‐analysis were LVEF, left ventricular global longitudinal strain (LV‐GLS), and LV‐global circumferential strain (GCS). Right ventricular indices were not analyzed due to insufficient reporting.

### 3.5 LVEF

#### 3.5.1 Mean Difference

**FIGURE 2:**
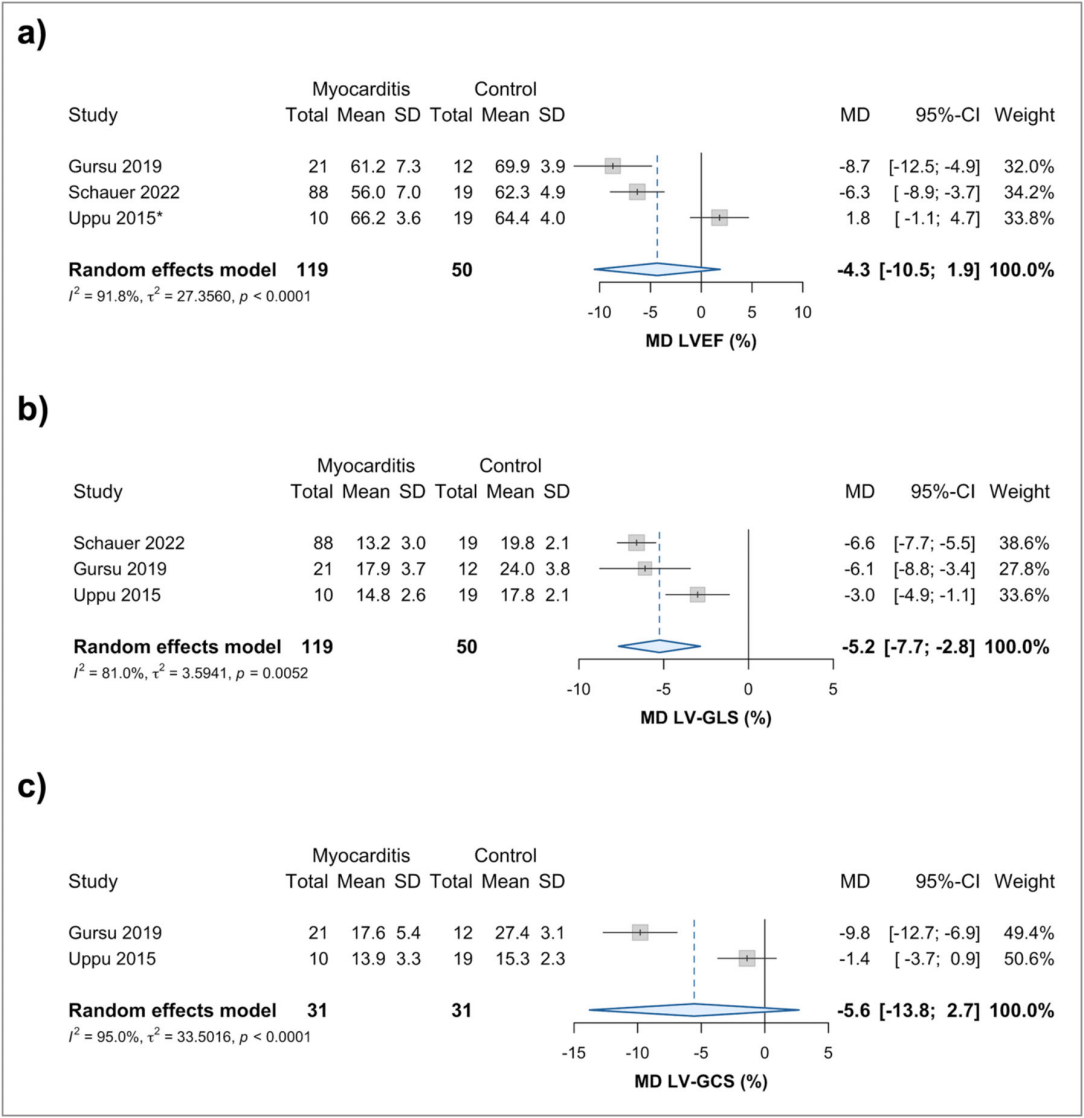
Forest plots for the mean difference in (a) LVEF, (b) LV‐GLS, and (c) LV‐GCS values between pediatric myocarditis patients and healthy controls. Only LV‐GLS reliably differentiates between myocarditis and healthy states. Strain values have been made absolute to aid in interpretation of mean differences. * Indicates studies in which median to mean imputation was used. CI, confidence interval; LVEF, left ventricular ejection fraction; LV‐GCS, left ventricular global circumferential strain; LV‐GLS, left ventricular global longitudinal strain; MD, mean difference; SD, standard deviation.

All three case–control studies reported LVEF. The pooled LVEF MD between pediatric myocarditis and healthy subjects was −4.3% (95% CI −10.5% to 1.9%, Figure 2a). Expectedly, the LVEF MD increased when data from Uppu et al. (who only included subjects with normal LVEF) was excluded (MD −7.1%, 95% CI −9.3% to −4.9%, *p* < 0.001, Figures S1, S2). An exploratory meta‐regression showed no effect of age on the LVEF MD between myocarditis and healthy subjects (*p* = 0.460, Figure S3).

#### 3.5.2 Pooled LVEF

**FIGURE 3:**
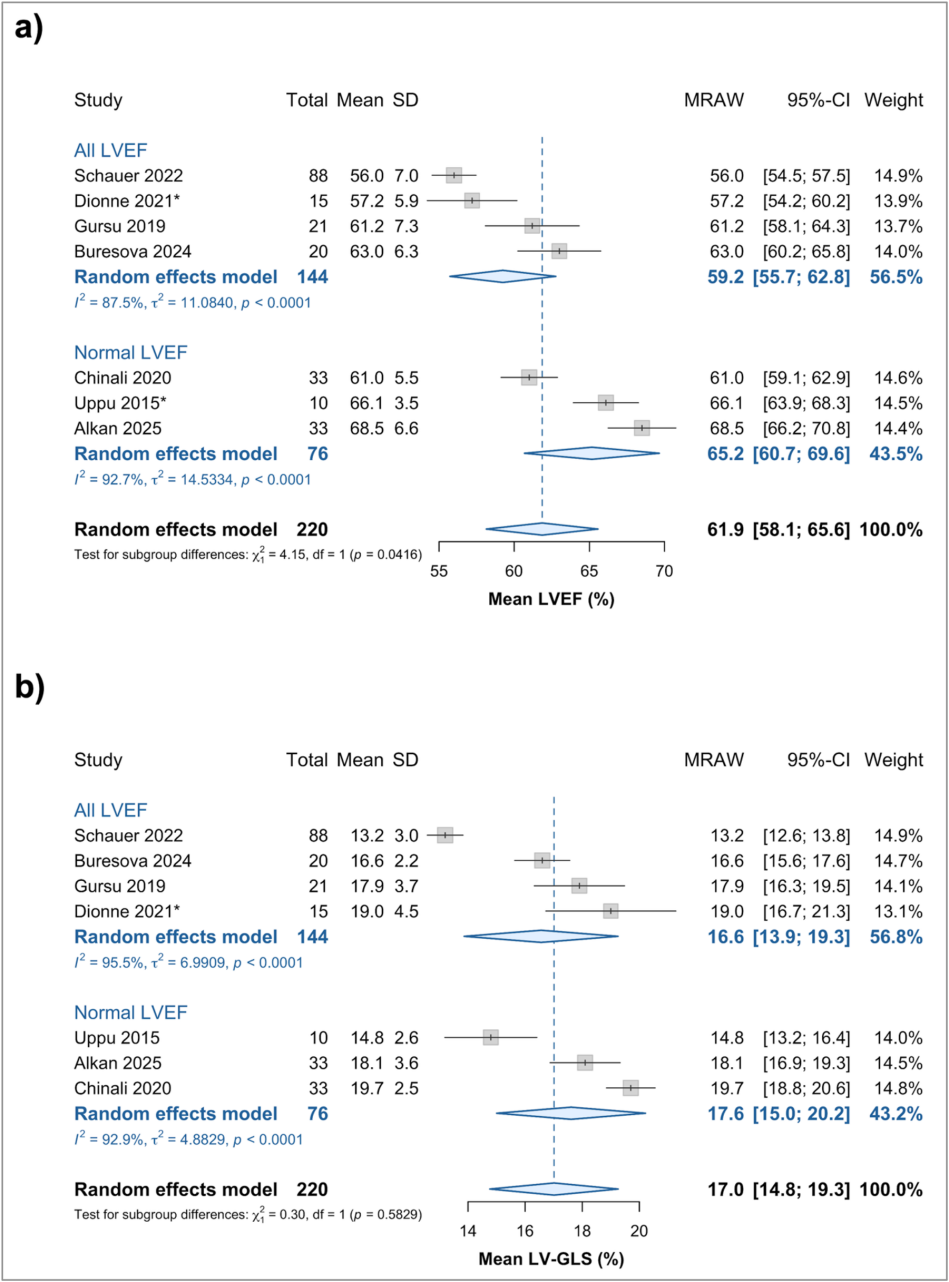
Forest plots and subgroup analyses for the pooled mean (a) LVEF and (b) LV‐GLS estimates in pediatric myocarditis. Although in pediatric myocarditis LVEF may not differ from normal values for this age group, LV‐GLS is substantially reduced. Importantly, LV‐GLS remains reduced across a range of LVEF values, thus potentially unmasking subclinical disease. * Indicates studies in which median to mean imputation was used. CI, confidence interval; LVEF, left ventricular ejection fraction; LV‐GLS, left ventricular global longitudinal strain; MRAW, untransformed mean; SD, standard deviation.

Data for myocarditis LVEF was available from all seven included studies. The mean pooled LVEF for pediatric myocarditis patients was 61.9% (95% CI 58.1% to 65.6%, Figure 3a). Subgroup analysis confirmed that mean LVEF differed significantly (*p* = 0.042) between studies specifying inclusion of patients with normal LVEF only (65.2%, 95% CI 60.7% to 69.6%) and those that did not (59.2%, 95% CI 55.7% to 62.8%, Figure 3a, Figure S4), even though both of these fall broadly within the accepted range [38]. An exploratory meta‐regression of the mean LVEF against mean age was statistically significant only when including neonatal and toddler age groups (*n* studies = 2, *p* < 0.001 in both linear and nonlinear analyses, Figures S5–S9). Exploratory sensitivity analysis was positive for the effect of myocarditis etiology (*p* = 0.037, Figures S10, S11), but this is likely underpowered.

### 3.6 LV‐GLS

#### 3.6.1 Mean‐Difference

All three case–control studies also reported on LV‐GLS. There was a statistically significant LV‐GLS pooled MD between myocarditis and healthy subjects (MD −5.2%, 95% CI −7.7% to −2.8%, Figure 2b). On sensitivity analysis, despite a statistically significant difference in LV‐GLS MD between Uppu et al. (who only included normal LVEF subjects) and studies reporting on the whole range of LVEF (*p* = 0.001), pooled MDs were consistently below 0 for all subgroups (normal LVEF: −3.0%, 95% CI −4.9% to −1.1%; all LVEF: −6.5%, 95% CI −7.6% to −5.5%, Figures S12, S13). An exploratory meta‐regression showed no effect of age on the LV‐GLS MD (*p* = 0.795, Figure S14).

#### 3.6.2 Pooled LV‐GLS

LV‐GLS data were available from all seven included studies. The mean pooled LV‐GLS for pediatric myocarditis patients was −17.0% (95% CI −19.3% to −14.8%, Figure 3b). In subgroup analysis, the mean LV‐GLS was not affected between groups of normal LVEF and all ranges of LVEF (*p* = 0.583, Figure 3b, Figure S15). An exploratory meta‐regression investigating the impact of study‐level mean LVEF on mean LV‐GLS was negative (*p* = 0.768, Figures S16, S17). Mean LV‐GLS was also not affected by the mean age once neonatal myocarditis subjects had been excluded (*p* > 0.05 in both linear and nonlinear analyses, Figures S18–S22). The etiology of myocarditis also had no statistically significant effect on the mean pooled LV‐GLS (*p* = 0.178, Figures S23, S24).

### 3.7 LV‐GCS

Only two case–control design studies reported left ventricular global circumferential strain (LV‐GCS). The LV‐GCS MD was not statistically significant between myocarditis and healthy subjects (MD −5.6%, 95% CI −13.8% to 2.7%, Figure 2c). The mean pooled LV‐GCS (from a total of three studies) was −18.4% (95% CI −23.7% to −13.0%, Figure S25). In exploratory subgroup analysis and meta‐regression, this was affected by LVEF status and the etiology of myocarditis (*p* = 0.039, *p* < 0.001, respectively, Figures S26–S28), but not by the mean age (*p* = 0.728, Figure S29), but these are all likely underpowered.

### 3.8 Left Ventricular Diastolic Indices

#### 3.8.1 LVEDD

Left ventricular end‐diastolic diameter (LVEDD) was the only diastolic echocardiographic index that could be meta‐analyzed in the present report. Only one case–control design study reported this endpoint hence only the pediatric myocarditis population mean pooled LVEDD is presented here. The pooled mean LVEDD from three pediatric myocarditis cohorts was 44 mm (95% CI 42–46 mm, Figure S30, S31).

### 3.9 Cardiac Magnetic Resonance

Out of the seven included studies, six also investigated their subjects using CMR. A meta‐analysis of correlations between STE and CMR parameters could not be conducted due to heterogenous statistical reporting by the individual studies. Hence, only main CMR findings, as well as concordance with STE, are synthesized in Table 2.

**TABLE 2:** CMR analysis of myocardial segment and layer involvement in pediatric myocarditis, as well as concordance between CMR and STE.

Report	CMR segmental/layer involvement	Reported CMR and STE comparison
Uppu et al. 2015 [26]	LGE was most often found in the basal, mid‐inferior, mid‐inferolateral, and mid‐anterolateral segments. CMR and STE strain overlap was better in the basal and mid‐ than in apical segments (18% vs. 35%, *p* = 0.006).	In 7/10 patients, LV‐GLS identified more abnormal segments than LGE CMR, but the overall correlation per patient was moderate (*r* = 0.68, *p* = 0.03).
Chinali et al. 2020 [29]	Edema most commonly occurred in the inferoseptal, inferior and inferolateral segments, which was consistent with the areas of LV‐GLS decline.	In the study, patients were split based on normal and reduced LV‐GLS which also differentiated their CMR edema by mass (*p* = 0.043) and by percentage (*p* = 0.041). Percentage of edema on CMR vs. LV‐GLS at admission was associated (*r* = 0.712, *p* = 0.01).
Dionne et al. 2021 [30]	LGE was present in 12/15 patients, and most often found in the inferolateral (*n* = 3) and anterolateral (*n* = 4) segments.	−
Schauer et al. 2022 [28]	LGE was most often found in basal, mid‐inferolateral, mid‐anterolateral, and apical‐lateral segments.	In the study, troponin‐positive patients were split based on CMR myocarditis positive and negative. LV‐GLS differentiated these groups from each other (*p* < 0.001) as well as from healthy controls. LV‐GLS cutoff of −17% yielded 90% sensitivity and 65% specificity in predicting positive CMR myocarditis in their cohort. A cutoff of −15.5% yielded 80% sensitivity and 85% specificity. Longitudinal strain was more abnormal in LGE positive than negative segments (*p* < 0.05).
Burešová et al. 2024 [31]	−	STE and CMR agreed varyingly across myocardial segments: Anterolateral (*κ* = 0.88) Inferolateral (*κ* = 0.57) Anteroseptal (*κ* = 0.41) Inferoseptal (*κ* = 0.40)
Alkan et al. 2025 [32]	LGE patterns were linear or patchy and mostly epicardial or mid‐wall. Only 2/33 patients had transmural involvement.	LV‐GLS cutoff of −18% yielded 52% sensitivity and 63% specificity in predicting myocarditis in their cohort. A cutoff of −16% yielded 40% sensitivity and 75% specificity. No association was observed between regional CMR edema and LV‐GLS < 18% (*p* = 0.732).

*Note*: STE reliably approximates the myocardial injury/dysfunction ascertained through the gold‐standard of CMR and may be a useful surrogate of this imaging modality.

Abbreviations: CMR, cardiac magnetic resonance; LGE, late gadolinium enhancement; LV‐GLS, left ventricular global longitudinal strain; STE, speckle‐tracking echocardiography.

## 4 Discussion

This systematic review and meta‐analysis suggests that LV‐GLS measured by STE reliably differentiates pediatric myocarditis from healthy controls (MD −5.2%, 95% CI −7.7% to −2.8%), even when ejection fraction is preserved. The pooled LV‐GLS across studies (−17.0%, 95% CI −19.3% to −14.8%) was lower than established pediatric reference values of −20.1% ± 0.3% and −19.8% ± 0.6% for children aged 10–14 years and adolescents aged 15–19 years, respectively [35]. We also confirm a strong and consistent relationship between STE strain abnormalities and the tissue‐level pathology identified by CMR. These findings emphasize the limited sensitivity of conventional echocardiographic indices to early or regional myocardial dysfunction and reinforce the role of strain imaging as an earlier diagnostic marker.

Importantly, we show that between subgroups of normal LVEF subjects only, and those representing all ranges of LVEF, there is an expected difference in the pooled mean LVEF (*p* = 0.042) but that the mean pooled LV‐GLS from these subgroups does not differ (*p* = 0.583). Even in subclinical myocarditis with preserved LVEF (mean 65.2%, 95% CI 58.1% to 65.6%), LV‐GLS is still reduced (mean −17.6%, 95% CI −20.2% to −15.0%) relative to the reference ranges mentioned above. Furthermore, despite a statistically significant difference in the LV‐GLS MD between normal‐ and all‐LVEF (*p* = 0.001), all the pooled MDs were clinically significant (MD and CI always < 0) and reliably differentiated myocarditis from healthy subjects.

In contrast, pooled data for LV‐GCS did not differentiate between myocarditis and controls. However, this is likely an underpowered conclusion, as only two studies were quantitively synthesized for this endpoint, rather than a commentary on the utility of LV‐GCS.

The overall superior ability of strain imaging to detect subclinical injury may be explained by myocardial fiber architecture and the regional injury common in early myocarditis [39]. LVEF is a crude, load‐dependent, volumetric measure of global pump function, and preferentially reflects the circumferential and radial component of myocardial contraction [12]. However, in focal myocarditis, inflammation and edema most frequently involve the subepicardial or mid‐wall layers, particularly in inferolateral and anterolateral segments [3, 26, 28], and lead to subclinical impairment in longitudinal and circumferential deformation. Therefore, unlike conventional TTE metrics that are affected by geometry and loading conditions, STE‐derived strain examines the myocardium itself and can pick up layer‐specific injury before cardiac insult becomes transmural and contributes to global systolic dysfunction.

### 4.1 Concordance Between STE and CMR

CMR‐derived strain is already known to correlate with CMR‐derived tissue characterization [40]. Our systematic review of the included studies provides strong evidence that STE findings are also a direct reflection of this tissue injury observed on CMR. Multiple studies found that the LV segments most affected by LGE or edema (basal‐ and mid‐inferolateral, and anterolateral walls) co‐localized with those exhibiting the most significant reduction in regional longitudinal strain [26, 28, 31]. Schauer et al. go further and demonstrate that regional strain within LGE‐positive segments was significantly worse than in LGE‐negative segments within the same patient [28]. Global strain reductions differentiated between CMR myocarditis‐positive and ‐negative patients [28] whilst also correlating with CMR edema burden (*r* ∼ 0.7) [26, 29]. This correlation was present in two of three studies which only investigated normal LVEF patients [26, 29, 32]; the lack of concordance in one study may have been due to the unaffected global systolic function but also due to difficulties in image acquisition, as described by the authors [32]. Nevertheless, the majority of the evidence points to STE as a reliable surrogate of the myocardial injury detected by CMR, and this has also been observed in the adult population [41].

Considering these encouraging findings in support of STE as an imaging alternative, the inherent drawbacks of CMR‐guided diagnosis become even more pronounced. Despite CMR being the reference standard for non‐invasive tissue characterization [4], its practical utility is frequently affected by logistical and clinical constraints [10, 11]. It remains a high‐cost, and resource‐intensive modality, often restricted to specialist centers with potentially extended reporting times [42]. This can translate to diagnostic delays, which become particularly consequential in the context of acute myocarditis. The relative clinical instability of pediatric patients further compounds this problem. Critically ill children often cannot tolerate prolonged acquisition times in the supine position or the risks associated with sedation or anesthesia [43].

Consequently, the reliance on CMR may represent a sub‐optimal diagnostic pathway. By validating STE as a robust surrogate of CMR‐derived tissue pathology, we highlight the potential use of STE as a low‐cost, rapidly accessible, and immediately interpretable bedside imaging tool.

### 4.2 Diagnosis and Prognosis

Compared to the adult meta‐analysis [18], which found that both LV‐GLS and LVEF were significantly impaired in myocarditis patients compared to controls, our pediatric analysis did not demonstrate such an effect for LVEF. This varied utility of LVEF most likely does not reflect a pathophysiological difference between pediatric and adult compensatory mechanisms but, more simply, shows selection bias. Patients included in the studies that formed part of the meta‐analysis by Khanna et al. may have presented later than those in the present report. It is well known that advanced myocarditis cases have very clearly reduced LVEF, and this echocardiographic parameter remains reliable for more decompensated and clinically symptomatic patients who may be in an intensive care unit [44]. However, when investigating earlier/milder cases, strain measurements begin to unmask what standard ejection fraction cannot, as outlined above. This may also extend into prognostication.

The clinical implications of these findings are significant. STE, and specifically LV‐GLS, should form part of the initial diagnostic work‐up for any child with suspected myocarditis. In the common and ambiguous scenario of a child presenting with compatible symptoms, elevated troponin and a normal LVEF, an abnormal (reduced) LV‐GLS should raise concerns. The −16% to −18% LV‐GLS range is emerging as a critical threshold for accurate diagnosis of myocarditis in both pediatric and adult studies [18, 28, 32, 45]. This is in accordance with the pooled LV‐GLS of −17.0% from the present meta‐analysis.

However, what we do not yet know is if there is concordance between increasingly impaired strain parameters and the severity of disease or outcomes. Hence, it would be important to not only examine the accuracy of diagnostic strain thresholds in larger, more heterogeneous cohorts, but also to regress strain parameters against heavily stratified/continuous demographic metrics at presentation and follow‐up, using rigorous and standardized imaging protocols. This would better characterize the STE per‐unit change effects on the likelihood of a positive diagnosis as well as on future risk and survival in various subgroups. Encouragingly, there is growing evidence supporting the prognostic value of strain. LV‐GLS, derived either via STE or CMR methods, has been associated with arrhythmia risk, major adverse cardiac events and residual dysfunction after recovery in patients with myocarditis [41, 46, 47, 48]. This aligns with new adult prognostic scores that incorporate LV‐GLS as a key variable for risk stratification [49]. Further research is warranted to determine the prognostic value of particular strain ranges in the context of the clinical condition and management approach.

Importantly, it should be acknowledged that technical and physiological factors can affect the reproducibility and interpretation of STE. Inter‐vendor, and inter‐ and intra‐observer variability, whilst generally good and smaller in comparison to conventional echocardiography, can still significantly alter observed strain values [50, 51, 52]. This variability is independent of the impact sonographer experience and image optimization may have, for example the effect of frame rate [53]. Interestingly, anterior chest wall deformities can also alter the measurement of myocardial strain [54]. Taken together, these factors highlight the potential variability in strain measurements and the challenges in reproducible interpretation of STE across pediatric populations. The standardization of imaging guidelines can help to solve these problems [50].

### 4.3 Influence of Etiology and Age

Overall, our meta‐regression found no effect of myocarditis etiology (viral/idiopathic vs. vaccine‐associated) on pooled LV‐GLS. However, this finding was likely underpowered, with only one study in the vaccine‐associated group [30], and should be interpreted with caution. In fact, vaccine‐related cases tend to show milder strain impairment [55] and therefore this remains an important area for future research.

After the exclusion of the neonatal and infant population, exploratory meta‐regression revealed no consistent effect of age on LV‐GLS. This suggests that strain impairment reflects pathology rather than developmental variation.

### 4.4 Limitations

Several limitations warrant consideration. Firstly, the number of included pediatric studies was small, with only three true case–control designs and a predominance of small, single‐center cohorts mostly exclusive of fulminant or severely impaired cases. Secondly, the exploratory sub‐analyses may have been underpowered due to the small number of available studies, and their findings should therefore be interpreted as hypothesis‐generating only. In given subgroup analyses stratified by LVEF, normal versus purely abnormal could not be accurately delineated due to no constituent studies specifying an inclusion criterion for only abnormal LVEF subjects; rather, all ranges of LVEF were examined against normal LVEF only. Thirdly, heterogeneity in imaging acquisition, strain software, and patient selection may introduce methodological variability and potential ecological bias. Finally, publication bias toward positive results cannot be excluded, given the small evidence base and observational design of most studies.

## 5 Conclusion

STE‐derived LV‐GLS appears to be a powerful and sensitive tool for detecting myocardial dysfunction in pediatric acute myocarditis, outperforming conventional LVEF. Its correlation with CMR findings supports its role as a complementary or even alternative diagnostic tool, particularly where CMR is impractical. Therefore, LV‐GLS could play a central role in the work‐up of pediatric patients suspected of having myocarditis.

## Funding

The authors declare that no funds, grants, or other support were received during the preparation of this manuscript.

## Conflicts of Interest

The authors declare no conflicts of interest.

## Supporting information




**Supporting Information File S1**: echo70443‐sup‐0001‐SuppMat.pdf

## Data Availability

The data that support the findings of this study are available from the corresponding author upon reasonable request.
