# Supplementary material for: The Utility of Strain Echocardiography in the Diagnosis of Pediatric Myocarditis: A Systematic Review and Meta‐Analysis
Source: Echocardiography. 2026 Apr 15;43:e70443. doi: 10.1111/echo.70443 (PMC13082184; doi:10.1111/echo.70443)
Supplement: Supplementary file 1 — Supporting Information File S1: echo70443‐sup‐0001‐SuppMat.pdf [file ECHO-43-e70443-s001.pdf]

The utility of strain echocardiography in the diagnosis of paediatric myocarditis: a systematic review and meta-analysis

Supplementary Appendix

Table of Contents

SUPPLEMENTARY METHODS 4

Search strategy 4
MEDLINE 4
Embase 4
Scopus 5

Sub-cohorts 5

SUPPLEMENTARY RESULTS 6

PRISMA checklist 6
Supplementary Table S1: PRISMA checklist 6

Study quality 9
Supplementary Table S2: QUADAS-2 results 9

LVEF 10
Mean difference 10
Supplementary Figure S1: Subgroup forest plot MD LVEF vs LVEF status 10
Supplementary Figure S2: Subgroup meta-analysis MD LVEF vs LVEF status model parameters 10
Supplementary Figure S3: MD LVEF versus age meta-regression (linear) model parameters 11
Pooled LVEF 11
Supplementary Figure S4: Subgroup meta-analysis mean LVEF vs LVEF status model parameters 11
Supplementary Figure S5: Forest plot mean LVEF, including Bigg et al. and Bhatia et al. 12
Supplementary Figure S6: Mean LVEF, including Bigg et al. and Bhatia et al., versus age meta-regression (linear) model parameters 13
Supplementary Figure S7: Mean LVEF, including Bigg et al. and Bhatia et al., versus age meta-regression (non-linear) model parameters 13
Supplementary Figure S8: Mean LVEF versus age meta-regression (linear) model parameters 14
Supplementary Figure S9: Mean LVEF versus age meta-regression (non-linear) model parameters 14
Supplementary Figure S10: Subgroup forest plot mean LVEF vs aetiology 15
Supplementary Figure S11: Subgroup meta-analysis mean LVEF vs aetiology model parameters 15

LV-GLS 16
Mean-difference 16
Supplementary Figure S12: Subgroup forest plot MD LV-GLS vs LVEF status 16
Supplementary Figure S13: Subgroup meta-analysis MD LV-GLS vs LVEF status model parameters 16
Supplementary Figure S14: MD LV-GLS versus age meta-regression (linear) model parameters 17
Pooled LV-GLS 17
Supplementary Figure S15: Subgroup meta-analysis mean LV-GLS vs LVEF status model parameters 17
Supplementary Figure S16: Mean LV-GLS versus mean LVEF meta-regression (linear) model parameters 18
Supplementary Figure S17: Mean LV-GLS versus mean LVEF meta-regression (non-linear) model parameters 18
Supplementary Figure S18: Forest plot mean LV-GLS, including Bigg et al. 19
Supplementary Figure S19: Mean LV-GLS, including Bigg et al., versus age meta-regression (linear) model parameters 20
Supplementary Figure S20: Mean LV-GLS, including Bigg et al., versus age meta-regression (non-linear) model parameters 20
Supplementary Figure S21: Mean LV-GLS versus age meta-regression (linear) model parameters 21

Supplementary Figure S22: Mean LV-GLS versus age meta-regression (non-linear) model parameters 21
Supplementary Figure S23: Subgroup forest plot mean LV-GLS vs aetiology 22
Supplementary Figure S24: Subgroup meta-analysis mean LV-GLS vs aetiology model parameters 22

LV-GCS **23**
Pooled LV-GCS 23
Supplementary Figure S25: Subgroup forest plot mean LV-GCS vs LVEF status 23
Supplementary Figure S26: Subgroup meta-analysis mean LV-GCS vs LVEF status model parameters 23
Supplementary Figure S27: Subgroup forest plot mean LV-GCS vs aetiology 24
Supplementary Figure S28: Subgroup meta-analysis mean LV-GCS vs aetiology model parameters 24
Supplementary Figure S29: Mean LV-GCS, versus age meta-regression (linear) model parameters 25

LVEDD **26**
Pooled LVEDD 26
Supplementary Figure S30: Forest plot mean LVEDD 26
Supplementary Figure S31: Mean LVEDD versus age meta-regression (linear) model parameters 26

Supplementary Methods

Search strategy

MEDLINE

1 global longitudinal strain.mp. or exp Global Longitudinal Strain/
2 strain echocardiography.mp.
3 (speckle-track* or speckle-tracking or speckle NEXT tracking or speckle NEXT track*).mp. [mp=title, book title, abstract, original title, name of substance word, subject heading word, floating sub-heading word, keyword heading word, organism supplementary concept word, protocol supplementary concept word, rare disease supplementary concept word, unique identifier, synonyms, population supplementary concept word, anatomy supplementary concept word]
4 (longitudinal or circumferential or radial or speckle or tracking or global or regional).mp.
5 strain.mp.
6 echocardiography.mp. or exp Echocardiography/
7 (gls or global longitudinal strains or lv gls or left ventricular global longitudinal strain or left ventricular global longitudinal strains or rv gls or right ventricular global longitudinal strain or right ventricular global longitudinal strains).mp.
8 3 and 6
9 4 and 5
10 1 or 2 or 7 or 8 or 9
11 myocarditis.mp. or exp Myocarditis/
12 (myocarditides or carditis or carditides or myocardic).mp.
13 11 or 12
14 10 and 13

Embase

1 Global Longitudinal Strain.mp. or exp global longitudinal strain/
2 exp speckle tracking echocardiography/ or exp three dimensional speckle tracking echocardiography/ or exp two dimensional speckle tracking echocardiography/
3 (speckle-tracking or speckle-track* or speckle NEXT tracking or speckle NEXT track*).mp.
4 (longitudinal or circumferential or radial or speckle or tracking or global or regional).mp. [mp=title, abstract, heading word, drug trade name, original title, device manufacturer, drug manufacturer, device trade name, keyword heading word, floating subheading word, candidate term word]
5 strain.mp.
6 echocardiography.mp. or exp echocardiography/
7 (gls or global longitudinal strains or lv gls or left ventricular global longitudinal strain or left ventricular global longitudinal strains or rv gls or right ventricular global longitudinal strain or right ventricular global longitudinal strains).mp. [mp=title, abstract, heading word, drug trade name, original title, device manufacturer, drug manufacturer, device trade name, keyword heading word, floating subheading word, candidate term word]
8 3 and 6
9 4 and 5
10 1 or 2 or 7 or 8 or 9
11 myocarditis.mp. or exp myocarditis/
12 (myocarditides or carditis or carditides or myocardic).mp.
13 11 or 12
14 10 and 13

Scopus

((TITLE-ABS-KEY ("Global Longitudinal Strain") OR INDEXTERMS ("global longitudinal strain")) OR (INDEXTERMS ("speckle tracking echocardiography") OR INDEXTERMS ("three dimensional speckle tracking echocardiography") OR INDEXTERMS ("two dimensional speckle tracking echocardiography")) OR (TITLE-ABS-KEY (gls OR "global longitudinal strains" OR "lv gls" OR "left ventricular global longitudinal strain" OR "left ventricular global longitudinal strains" OR "rv gls" OR "right ventricular global longitudinal strain" OR "right ventricular global longitudinal strains")) OR ((TITLE-ABS-KEY (speckle-tracking OR speckle-track* OR speckle W/1 tracking OR speckle W/1 track*)) AND (TITLE-ABS-KEY (echocardiography) OR INDEXTERMS (echocardiography))) OR ((TITLE-ABS-KEY (longitudinal OR circumferential OR radial OR speckle OR tracking OR global OR regional)) AND (TITLE-ABS-KEY (strain)))) AND ((TITLE-ABS-KEY (myocarditis) OR INDEXTERMS (myocarditis)) OR (TITLE-ABS-KEY (myocarditides OR carditis OR carditides OR myocardic)))

Sub-cohorts

The below studies' patients were not analysed in full and only their myocarditis arms/subgroups were extracted and formed part of the quantitative analysis:

Schauer et al. 2022	of the 108 patients presenting with chest pain and elevated troponin, only data from those with CMR-positive myocarditis (n = 88) was used.
Bhatia et al. 2025	of the MISC myocarditis (n = 52) and viral myocarditis (n = 23) arms, only data from the latter was used.

All other studies were extracted and analysed in full.

Supplementary Results

PRISMA checklist

Supplementary Table S1: PRISMA checklist

Section and Topic	Item #	Checklist item	Location where item is reported
**TITLE**			
Title	1	Identify the report as a systematic review.	Title
**ABSTRACT**			
Abstract	2	See the PRISMA 2020 for Abstracts checklist.	Abstract
**INTRODUCTION**			
Rationale	3	Describe the rationale for the review in the context of existing knowledge.	Introduction
Objectives	4	Provide an explicit statement of the objective(s) or question(s) the review addresses.	Introduction
**METHODS**			
Eligibility criteria	5	Specify the inclusion and exclusion criteria for the review and how studies were grouped for the syntheses.	Study eligibility
Information sources	6	Specify all databases, registers, websites, organisations, reference lists and other sources searched or consulted to identify studies. Specify the date when each source was last searched or consulted.	Protocol and search strategy
Search strategy	7	Present the full search strategies for all databases, registers and websites, including any filters and limits used.	Supplement
Selection process	8	Specify the methods used to decide whether a study met the inclusion criteria of the review, including how many reviewers screened each record and each report retrieved, whether they worked independently, and if applicable, details of automation tools used in the process.	Study eligibility, Screening and extraction
Data collection process	9	Specify the methods used to collect data from reports, including how many reviewers collected data from each report, whether they worked independently, any processes for obtaining or confirming data from study investigators, and if applicable, details of automation tools used in the process.	Screening and extraction
Data items	10a	List and define all outcomes for which data were sought. Specify whether all results that were compatible with each outcome domain in each study were sought (e.g. for all measures, time points, analyses), and if not, the methods used to decide which results to collect.	Outcomes
	10b	List and define all other variables for which data were sought (e.g. participant and intervention characteristics, funding sources). Describe any assumptions made about any missing or unclear information.	Outcomes, Statistics
Study risk of bias assessment	11	Specify the methods used to assess risk of bias in the included studies, including details of the tool(s) used, how many reviewers assessed each study and whether they worked independently, and if applicable, details of automation tools used in the process.	Study quality appraisal
Effect measures	12	Specify for each outcome the effect measure(s) (e.g. risk ratio, mean difference) used in the synthesis or presentation of results.	Statistics

Section and Topic	Item #	Checklist item	Location where item is reported
Synthesis methods	13a	Describe the processes used to decide which studies were eligible for each synthesis (e.g. tabulating the study intervention characteristics and comparing against the planned groups for each synthesis (item #5)).	Outcomes, Statistics
	13b	Describe any methods required to prepare the data for presentation or synthesis, such as handling of missing summary statistics, or data conversions.	Statistics, Screening and extraction
	13c	Describe any methods used to tabulate or visually display results of individual studies and syntheses.	Statistics
	13d	Describe any methods used to synthesize results and provide a rationale for the choice(s). If meta-analysis was performed, describe the model(s), method(s) to identify the presence and extent of statistical heterogeneity, and software package(s) used.	Statistics
	13e	Describe any methods used to explore possible causes of heterogeneity among study results (e.g. subgroup analysis, meta-regression).	Statistics
	13f	Describe any sensitivity analyses conducted to assess robustness of the synthesized results.	Statistics
Reporting bias assessment	14	Describe any methods used to assess risk of bias due to missing results in a synthesis (arising from reporting biases).	NA
Certainty assessment	15	Describe any methods used to assess certainty (or confidence) in the body of evidence for an outcome.	Methods
**RESULTS**			
Study selection	16a	Describe the results of the search and selection process, from the number of records identified in the search to the number of studies included in the review, ideally using a flow diagram.	Search results
	16b	Cite studies that might appear to meet the inclusion criteria, but which were excluded, and explain why they were excluded.	Search results
Study characteristics	17	Cite each included study and present its characteristics.	Table 1
Risk of bias in studies	18	Present assessments of risk of bias for each included study.	Supplement
Results of individual studies	19	For all outcomes, present, for each study: (a) summary statistics for each group (where appropriate) and (b) an effect estimate and its precision (e.g. confidence/credible interval), ideally using structured tables or plots.	Table 1, Fig. 2, Fig. 3
Results of syntheses	20a	For each synthesis, briefly summarise the characteristics and risk of bias among contributing studies.	Results
	20b	Present results of all statistical syntheses conducted. If meta-analysis was done, present for each the summary estimate and its precision (e.g. confidence/credible interval) and measures of statistical heterogeneity. If comparing groups, describe the direction of the effect.	Results, Supplement
	20c	Present results of all investigations of possible causes of heterogeneity among study results.	Results, Supplement
	20d	Present results of all sensitivity analyses conducted to assess the robustness of the synthesized results.	Results, Supplement
Reporting biases	21	Present assessments of risk of bias due to missing results (arising from reporting biases) for each synthesis assessed.	NA
Certainty of	22	Present assessments of certainty (or confidence) in the body of evidence for each outcome assessed.	Results, Supplement

Section and Topic	Item #	Checklist item	Location where item is reported
evidence			
**DISCUSSION**			
Discussion	23a	Provide a general interpretation of the results in the context of other evidence.	Discussion
	23b	Discuss any limitations of the evidence included in the review.	Discussion
	23c	Discuss any limitations of the review processes used.	Limitations
	23d	Discuss implications of the results for practice, policy, and future research.	Discussion, Diagnosis and prognosis
**OTHER INFORMATION**			
Registration and protocol	24a	Provide registration information for the review, including register name and registration number, or state that the review was not registered.	Protocol and search strategy
	24b	Indicate where the review protocol can be accessed, or state that a protocol was not prepared.	Protocol and search strategy
	24c	Describe and explain any amendments to information provided at registration or in the protocol.	Protocol and search strategy
Support	25	Describe sources of financial or non-financial support for the review, and the role of the funders or sponsors in the review.	NA
Competing interests	26	Declare any competing interests of review authors.	None
Availability of data, code and other materials	27	Report which of the following are publicly available and where they can be found: template data collection forms; data extracted from included studies; data used for all analyses; analytic code; any other materials used in the review.	Study data publicly available, synthesis material and code available upon reasonable request

From: Page MJ, McKenzie JE, Bossuyt PM, Boutron I, Hoffmann TC, Mulrow CD, et al. The PRISMA 2020 statement: an updated guideline for reporting systematic reviews. BMJ 2021;372:n71. doi: 10.1136/bmj.n71. This work is licensed under CC BY 4.0. To view a copy of this license, visit https://creativecommons.org/licenses/by/4.0/

Study quality

Supplementary Table S2: QUADAS-2 results

Report	Risk of bias				Applicability concerns		
	Patient selection	Index test	Reference standard	Flow and timing	Patient selection	Index test	Reference standard
**Uppu et al. 2015**	Low	Low	Low	Low	Low	Low	Low
**Gursu et al. 2019**	High	Low	Low	Low	Low	Low	Low
**Schauer et al. 2022**	Low	Low	Low	Low	Low	Low	Low
**Chinali et al. 2020**	Low	Low	Low	Low	Low	Low	Low
**Dionne et al. 2021**	Low	Low	Low	Low	Low	Low	Low
**Buresova et al. 2024**	Low	Low	Low	Low	Low	Low	Low
**Alkan et al. 2025**	Low	Low	Low	Low	Low	Low	Low
Bigg et al. 2023	*Low*	*Low*	*Low*	*Low*	*Low*	*Low*	*Low*
Bhatia et al. 2025	*Low*	*Low*	*Low*	*Low*	*Low*	*Low*	*Low*

LVEF

Mean difference

Supplementary Figure S1: Subgroup forest plot MD LVEF vs LVEF status

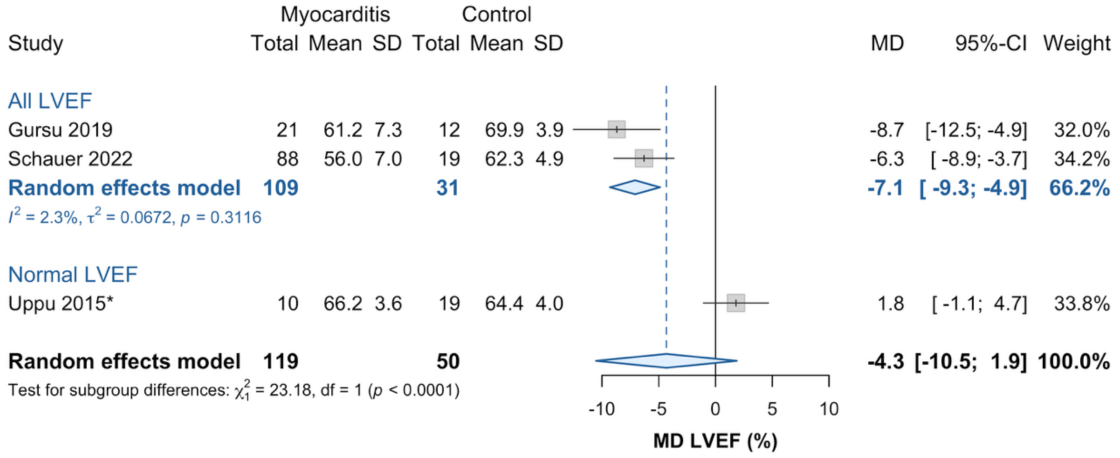

Supplementary Figure S2: Subgroup meta-analysis MD LVEF vs LVEF status model parameters

```
                    MD             95%-CI %W(random)    lvef_norm
Gursu 2019    -8.7000 [-12.5232; -4.8768]       32.0     All LVEF
Schauer 2022 -6.3000 [ -8.9445; -3.6555]       34.2     All LVEF
Uppu 2015*    1.8000 [ -1.0659;  4.6659]       33.8 Normal LVEF

Number of studies: k = 3
Number of observations: o = 169 (o.e = 119, o.c = 50)

                            MD              95%-CI     z p-value
Random effects model -4.3292 [-10.5188; 1.8604] -1.37  0.1704

Quantifying heterogeneity (with 95%-CIs):
 tau^2 = 27.3560 [5.5941; >274.2403]; tau = 5.2303 [2.3652; >16.5602]
 I^2 = 91.8% [79.2%; 96.8%]; H = 3.49 [2.19; 5.57]

Test of heterogeneity:
     Q d.f.  p-value
 24.41    2 < 0.0001

Results for subgroups (random effects model):
                          k      MD             95%-CI  tau^2    tau    Q  I^2
lvef_norm = All LVEF      2 -7.0865 [-9.2945; -4.8786] 0.0672 0.2592 1.02 2.3%
lvef_norm = Normal LVEF   1  1.8000 [-1.0659;  4.6659]     --     -- 0.00   --

Test for subgroup differences (random effects model):
                   Q d.f.  p-value
Between groups 23.18    1 < 0.0001

Details of meta-analysis methods:
- Inverse variance method
- DerSimonian-Laird estimator for tau^2
- Jackson method for confidence interval of tau^2 and tau
- Calculation of I^2 based on Q
```

Supplementary Figure S3: MD LVEF versus age meta-regression (linear) model parameters

```
Mixed-Effects Model (k = 3; tau^2 estimator: DL)

  logLik  deviance       AIC       BIC      AICc
 -8.7553    9.3015   23.5105   20.8063   47.5105

tau^2 (estimated amount of residual heterogeneity):     36.5023 (SE = 54.4617)
tau (square root of estimated tau^2 value):             6.0417
I^2 (residual heterogeneity / unaccounted variability): 94.79%
H^2 (unaccounted variability / sampling variability):   19.18
R^2 (amount of heterogeneity accounted for):            0.00%

Test for Residual Heterogeneity:
QE(df = 1) = 19.1788, p-val < .0001

Test of Moderators (coefficient 2):
QM(df = 1) = 0.5475, p-val = 0.4593

Model Results:

         estimate       se     zval    pval     ci.lb    ci.ub
intrcpt  -27.1305  31.0040  -0.8751  0.3815  -87.8972  33.6362
age_all    1.5782   2.1329   0.7399  0.4593   -2.6022   5.7586

---
Signif. codes:  0 ‘***’ 0.001 ‘**’ 0.01 ‘*’ 0.05 ‘.’ 0.1 ‘ ’ 1
```

Pooled LVEF

Supplementary Figure S4: Subgroup meta-analysis mean LVEF vs LVEF status model parameters

```
                 mean             95%-CI %W(random)   lvef_norm
Schauer 2022  56.0000 [54.5375; 57.4625]       14.9    All LVEF
Dionne 2021*  57.2000 [54.2142; 60.1858]       13.9    All LVEF
Chinali 2020  61.0000 [59.1235; 62.8765]       14.6 Normal LVEF
Gursu 2019    61.2000 [58.0778; 64.3222]       13.7    All LVEF
Buresova 2024 63.0000 [60.2390; 65.7610]       14.0    All LVEF
Uppu 2015*    66.1000 [63.9307; 68.2693]       14.5 Normal LVEF
Alkan 2025    68.5000 [66.2482; 70.7518]       14.4 Normal LVEF

Number of studies: k = 7
Number of observations: o = 220

                        mean             95%-CI
Random effects model 61.8568 [58.1458; 65.5679]

Quantifying heterogeneity (with 95%-CIs):
 tau^2 = 23.5629 [7.7553; 109.1802]; tau = 4.8542 [2.7848; 10.4489]
 I^2 = 94.9% [91.7%; 96.8%]; H = 4.42 [3.46; 5.63]

Test of heterogeneity:
      Q d.f.  p-value
 116.98    6 < 0.0001

Results for subgroups (random effects model):
                          k    mean             95%-CI   tau^2    tau     Q   I^2
lvef_norm = All LVEF      4 59.2435 [55.7258; 62.7612] 11.0840 3.3293 24.08 87.5%
lvef_norm = Normal LVEF   3 65.1656 [60.6840; 69.6473] 14.5334 3.8123 27.47 92.7%

Test for subgroup differences (random effects model):
                  Q d.f. p-value
Between groups 4.15    1  0.0416

Details of meta-analysis methods:
- Inverse variance method
- DerSimonian-Laird estimator for tau^2
- Jackson method for confidence interval of tau^2 and tau
- Calculation of I^2 based on Q
- Untransformed (raw) means
```

Supplementary Figure S5: Forest plot mean LVEF, including Bigg et al. and Bhatia et al.

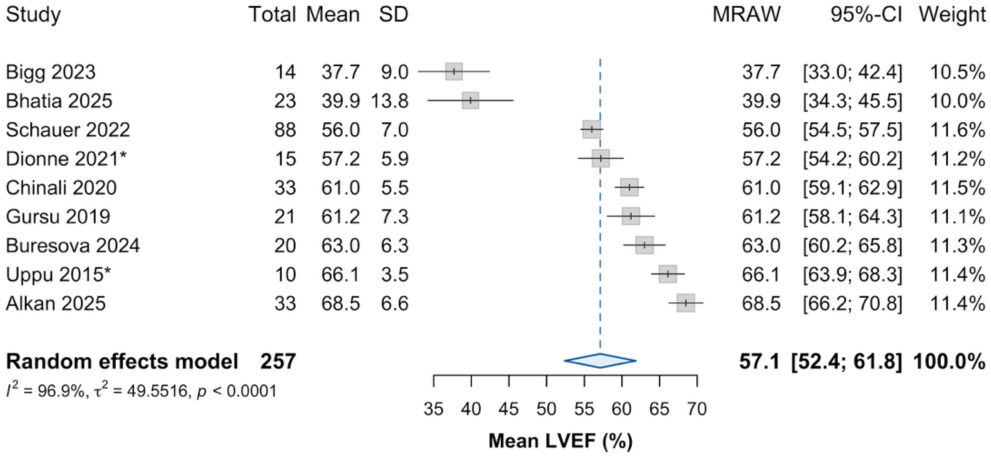

Supplementary Figure S6: Mean LVEF, including Bigg et al. and Bhatia et al., versus age meta-regression (linear) model parameters

```
Mixed-Effects Model (k = 9; tau^2 estimator: DL)

  logLik  deviance       AIC       BIC      AICc
-27.0121   31.3475   60.0241   60.6158   64.8241

tau^2 (estimated amount of residual heterogeneity):     32.5493 (SE = 20.8997)
tau (square root of estimated tau^2 value):             5.7052
I^2 (residual heterogeneity / unaccounted variability): 95.56%
H^2 (unaccounted variability / sampling variability):   22.53
R^2 (amount of heterogeneity accounted for):            34.31%

Test for Residual Heterogeneity:
QE(df = 7) = 157.6939, p-val < .0001

Test of Moderators (coefficient 2):
QM(df = 1) = 19.8440, p-val < .0001

Model Results:

                      estimate      se    zval    pval    ci.lb    ci.ub
intrcpt                37.5410  4.8548  7.7327  <.0001  28.0257  47.0563  ***
age_myocarditis_mean    1.6815  0.3775  4.4547  <.0001   0.9417   2.4214  ***

---
Signif. codes:  0 ‘***’ 0.001 ‘**’ 0.01 ‘*’ 0.05 ‘.’ 0.1 ‘ ’ 1
```

Supplementary Figure S7: Mean LVEF, including Bigg et al. and Bhatia et al., versus age meta-regression (non-linear) model parameters

```
Mixed-Effects Model (k = 9; tau^2 estimator: DL)

  logLik  deviance       AIC       BIC      AICc
-26.6669   30.6571   63.3337   64.3199   83.3337

tau^2 (estimated amount of residual heterogeneity):     34.4076 (SE = 25.7367)
tau (square root of estimated tau^2 value):             5.8658
I^2 (residual heterogeneity / unaccounted variability): 95.62%
H^2 (unaccounted variability / sampling variability):   22.86
R^2 (amount of heterogeneity accounted for):            30.56%

Test for Residual Heterogeneity:
QE(df = 5) = 114.2813, p-val < .0001

Test of Moderators (coefficients 2:4):
QM(df = 3) = 19.8051, p-val = 0.0002

Model Results:

                                   estimate       se    zval    pval    ci.lb    ci.ub
intrcpt                             34.9717   5.8351  5.9933  <.0001  23.5351  46.4082  ***
ns(age_myocarditis_mean, df = 3)1   20.0113   8.0184  2.4957  0.0126   4.2954  35.7271    *
ns(age_myocarditis_mean, df = 3)2   49.1921  17.2606  2.8500  0.0044  15.3619  83.0223   **
ns(age_myocarditis_mean, df = 3)3   14.0981   7.6223  1.8496  0.0644  -0.8414  29.0375    .

---
Signif. codes:  0 ‘***’ 0.001 ‘**’ 0.01 ‘*’ 0.05 ‘.’ 0.1 ‘ ’ 1
```

Supplementary Figure S8: Mean LVEF versus age meta-regression (linear) model parameters

```
Mixed-Effects Model (k = 7; tau^2 estimator: DL)

  logLik  deviance       AIC       BIC      AICc
-20.3928   25.6539   46.7856   46.6233   54.7856

tau^2 (estimated amount of residual heterogeneity):     29.2895 (SE = 21.0084)
tau (square root of estimated tau^2 value):             5.4120
I^2 (residual heterogeneity / unaccounted variability): 95.72%
H^2 (unaccounted variability / sampling variability):   23.38
R^2 (amount of heterogeneity accounted for):            0.00%

Test for Residual Heterogeneity:
QE(df = 5) = 116.8837, p-val < .0001

Test of Moderators (coefficient 2):
QM(df = 1) = 0.0266, p-val = 0.8705

Model Results:

                      estimate       se    zval    pval    ci.lb    ci.ub
intrcpt                59.1276  16.8774  3.5034  0.0005  26.0485  92.2066  ***
age_myocarditis_mean    0.1924   1.1803  0.1630  0.8705  -2.1209   2.5057

---
Signif. codes:  0 ‘***’ 0.001 ‘**’ 0.01 ‘*’ 0.05 ‘.’ 0.1 ‘ ’ 1
```

Supplementary Figure S9: Mean LVEF versus age meta-regression (non-linear) model parameters

```
Mixed-Effects Model (k = 7; tau^2 estimator: DL)

  logLik  deviance       AIC       BIC      AICc
-20.9361   26.7406   51.8722   51.6017  111.8722

tau^2 (estimated amount of residual heterogeneity):     43.4583 (SE = 39.4372)
tau (square root of estimated tau^2 value):             6.5923
I^2 (residual heterogeneity / unaccounted variability): 96.82%
H^2 (unaccounted variability / sampling variability):   31.49
R^2 (amount of heterogeneity accounted for):            0.00%

Test for Residual Heterogeneity:
QE(df = 3) = 94.4565, p-val < .0001

Test of Moderators (coefficients 2:4):
QM(df = 3) = 0.3365, p-val = 0.9530

Model Results:

                                   estimate       se     zval    pval     ci.lb    ci.ub
intrcpt                             61.1862   6.1442   9.9583  <.0001   49.1437  73.2287  ***
ns(age_myocarditis_mean, df = 3)1   -4.8101  10.4531  -0.4602  0.6454  -25.2978  15.6777
ns(age_myocarditis_mean, df = 3)2    4.5824  20.3738   0.2249  0.8220  -35.3494  44.5143
ns(age_myocarditis_mean, df = 3)3    1.2940   9.4418   0.1371  0.8910  -17.2116  19.7996

---
Signif. codes:  0 ‘***’ 0.001 ‘**’ 0.01 ‘*’ 0.05 ‘.’ 0.1 ‘ ’ 1
```

Supplementary Figure S10: Subgroup forest plot mean LVEF vs aetiology

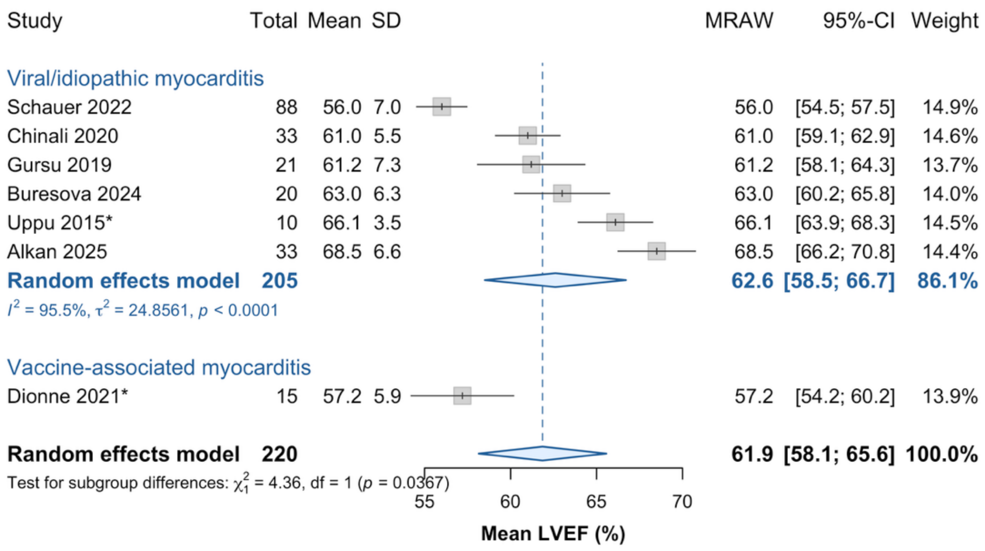

Supplementary Figure S11: Subgroup meta-analysis mean LVEF vs aetiology model parameters

```
                 mean             95%-CI %W(random)                     aetiology
Schauer 2022  56.0000 [54.5375; 57.4625]       14.9   Viral/idiopathic myocarditis
Dionne 2021*  57.2000 [54.2142; 60.1858]       13.9 Vaccine-associated myocarditis
Chinali 2020  61.0000 [59.1235; 62.8765]       14.6   Viral/idiopathic myocarditis
Gursu 2019    61.2000 [58.0778; 64.3222]       13.7   Viral/idiopathic myocarditis
Buresova 2024 63.0000 [60.2390; 65.7610]       14.0   Viral/idiopathic myocarditis
Uppu 2015*    66.1000 [63.9307; 68.2693]       14.5   Viral/idiopathic myocarditis
Alkan 2025    68.5000 [66.2482; 70.7518]       14.4   Viral/idiopathic myocarditis

Number of studies: k = 7
Number of observations: o = 220

                        mean             95%-CI
Random effects model 61.8568 [58.1458; 65.5679]

Quantifying heterogeneity (with 95%-CIs):
 tau^2 = 23.5629 [7.7553; 109.1802]; tau = 4.8542 [2.7848; 10.4489]
 I^2 = 94.9% [91.7%; 96.8%]; H = 4.42 [3.46; 5.63]

Test of heterogeneity:
      Q d.f.  p-value
 116.98    6 < 0.0001

Results for subgroups (random effects model):
                                              k    mean             95%-CI   tau^2    tau      Q   I^2
aetiology = Viral/idiopathic myocarditis      6 62.6070 [58.5064; 66.7076] 24.8561 4.9856 110.06 95.5%
aetiology = Vaccine-associated myocarditis    1 57.2000 [54.2142; 60.1858]      --     --   0.00    --

Test for subgroup differences (random effects model):
                  Q d.f. p-value
Between groups 4.36    1  0.0367

Details of meta-analysis methods:
- Inverse variance method
- DerSimonian-Laird estimator for tau^2
- Jackson method for confidence interval of tau^2 and tau
- Calculation of I^2 based on Q
- Untransformed (raw) means
```

LV-GLS

Mean-difference

Supplementary Figure S12: Subgroup forest plot MD LV-GLS vs LVEF status

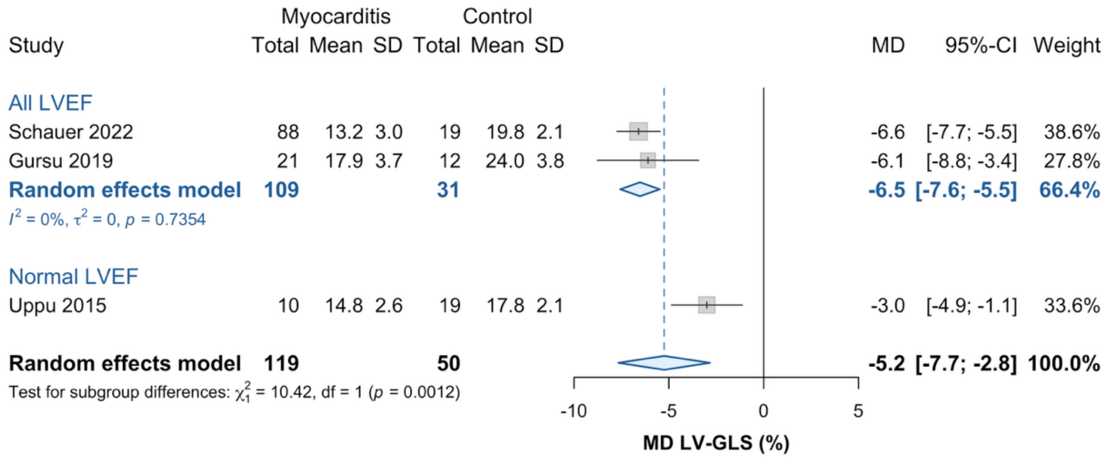

Supplementary Figure S13: Subgroup meta-analysis MD LV-GLS vs LVEF status model parameters

```
                   MD             95%-CI %W(random)    lvef_norm
Schauer 2022 -6.6000 [-7.7334; -5.4666]       38.6     All LVEF
Gursu 2019   -6.1000 [-8.7696; -3.4304]       27.8     All LVEF
Uppu 2015    -3.0000 [-4.8677; -1.1323]       33.6 Normal LVEF

Number of studies: k = 3
Number of observations: o = 169 (o.e = 119, o.c = 50)

                          MD             95%-CI     z  p-value
Random effects model -5.2498 [-7.6620; -2.8376] -4.27 < 0.0001

Quantifying heterogeneity (with 95%-CIs):
 tau^2 = 3.5941 [0.1581; >100.0000]; tau = 1.8958 [0.3977; >10.0000]
 I^2 = 81.0% [40.7%; 93.9%]; H = 2.30 [1.30; 4.06]

Test of heterogeneity:
     Q d.f. p-value
 10.54    2  0.0052

Results for subgroups (random effects model):
                          k      MD             95%-CI tau^2 tau    Q  I^2
lvef_norm = All LVEF      2 -6.5236 [-7.5669; -5.4804]     0   0 0.11 0.0%
lvef_norm = Normal LVEF   1 -3.0000 [-4.8677; -1.1323]    --  -- 0.00   --

Test for subgroup differences (random effects model):
                   Q d.f. p-value
Between groups 10.42    1  0.0012

Details of meta-analysis methods:
- Inverse variance method
- DerSimonian-Laird estimator for tau^2
- Jackson method for confidence interval of tau^2 and tau
- Calculation of I^2 based on Q
```

Supplementary Figure S14: MD LV-GLS versus age meta-regression (linear) model parameters

```
Mixed-Effects Model (k = 3; tau^2 estimator: DL)

  logLik  deviance       AIC       BIC      AICc
 -6.2369    7.5341   18.4739   15.7697   42.4739

tau^2 (estimated amount of residual heterogeneity):     6.2857 (SE = 9.8233)
tau (square root of estimated tau^2 value):             2.5071
I^2 (residual heterogeneity / unaccounted variability): 90.49%
H^2 (unaccounted variability / sampling variability):   10.52
R^2 (amount of heterogeneity accounted for):            0.00%

Test for Residual Heterogeneity:
QE(df = 1) = 10.5185, p-val = 0.0012

Test of Moderators (coefficient 2):
QM(df = 1) = 0.0677, p-val = 0.7947

Model Results:

         estimate       se     zval    pval     ci.lb    ci.ub
intrcpt   -8.7896  13.7310  -0.6401  0.5221  -35.7018  18.1227
age_all    0.2443   0.9386   0.2602  0.7947   -1.5954   2.0839

---
Signif. codes:  0 ‘***’ 0.001 ‘**’ 0.01 ‘*’ 0.05 ‘.’ 0.1 ‘ ’ 1
```

Pooled LV-GLS

Supplementary Figure S15: Subgroup meta-analysis mean LV-GLS vs LVEF status model parameters

```
                 mean             95%-CI %W(random)   lvef_norm
Schauer 2022  13.2000 [12.5732; 13.8268]       14.9    All LVEF
Uppu 2015     14.8000 [13.1885; 16.4115]       14.0 Normal LVEF
Buresova 2024 16.6000 [15.6358; 17.5642]       14.7    All LVEF
Gursu 2019    17.9000 [16.3175; 19.4825]       14.1    All LVEF
Alkan 2025    18.1000 [16.8717; 19.3283]       14.5 Normal LVEF
Dionne 2021*  19.0000 [16.7227; 21.2773]       13.1    All LVEF
Chinali 2020  19.7000 [18.8470; 20.5530]       14.8 Normal LVEF

Number of studies: k = 7
Number of observations: o = 220

                        mean             95%-CI
Random effects model 17.0116 [14.7667; 19.2566]

Quantifying heterogeneity (with 95%-CIs):
 tau^2 = 8.6852 [2.4525; 35.7704]; tau = 2.9471 [1.5660; 5.9808]
 I^2 = 96.6% [94.8%; 97.8%]; H = 5.41 [4.36; 6.70]

Test of heterogeneity:
      Q d.f.  p-value
 175.55    6 < 0.0001

Results for subgroups (random effects model):
                          k    mean             95%-CI  tau^2    tau     Q   I^2
lvef_norm = All LVEF      4 16.5594 [13.8671; 19.2517] 6.9909 2.6440 66.22 95.5%
lvef_norm = Normal LVEF   3 17.6090 [15.0051; 20.2128] 4.8829 2.2097 28.26 92.9%

Test for subgroup differences (random effects model):
                  Q d.f. p-value
Between groups 0.30    1  0.5829

Details of meta-analysis methods:
- Inverse variance method
- DerSimonian-Laird estimator for tau^2
- Jackson method for confidence interval of tau^2 and tau
- Calculation of I^2 based on Q
- Untransformed (raw) means
```

Supplementary Figure S16: Mean LV-GLS versus mean LVEF meta-regression (linear) model parameters

```
Mixed-Effects Model (k = 7; tau^2 estimator: DL)

  logLik  deviance       AIC       BIC      AICc
-15.7893   25.5304   37.5787   37.4164   45.5787

tau^2 (estimated amount of residual heterogeneity):     7.7167 (SE = 5.8635)
tau (square root of estimated tau^2 value):             2.7779
I^2 (residual heterogeneity / unaccounted variability): 95.58%
H^2 (unaccounted variability / sampling variability):   22.61
R^2 (amount of heterogeneity accounted for):            11.15%

Test for Residual Heterogeneity:
QE(df = 5) = 113.0307, p-val < .0001

Test of Moderators (coefficient 2):
QM(df = 1) = 0.0872, p-val = 0.7678

Model Results:

                      estimate       se    zval    pval     ci.lb    ci.ub
intrcpt                12.2722  16.0779  0.7633  0.4453  -19.2399  43.7842
lvef_myocarditis_mean   0.0765   0.2592  0.2952  0.7678   -0.4316   0.5846

---
Signif. codes:  0 ‘***’ 0.001 ‘**’ 0.01 ‘*’ 0.05 ‘.’ 0.1 ‘ ’ 1
```

Supplementary Figure S17: Mean LV-GLS versus mean LVEF meta-regression (non-linear) model parameters

```
Mixed-Effects Model (k = 7; tau^2 estimator: DL)

  logLik  deviance       AIC       BIC      AICc
-10.8314   15.6146   31.6629   31.3924   91.6629

tau^2 (estimated amount of residual heterogeneity):     1.5481 (SE = 1.7381)
tau (square root of estimated tau^2 value):             1.2442
I^2 (residual heterogeneity / unaccounted variability): 74.61%
H^2 (unaccounted variability / sampling variability):   3.94
R^2 (amount of heterogeneity accounted for):            82.18%

Test for Residual Heterogeneity:
QE(df = 3) = 11.8165, p-val = 0.0080

Test of Moderators (coefficients 2:4):
QM(df = 3) = 13.7109, p-val = 0.0033

Model Results:

                                    estimate      se     zval    pval     ci.lb    ci.ub
intrcpt                              14.1701  1.1444  12.3819  <.0001   11.9271  16.4131  ***
ns(lvef_myocarditis_mean, df = 3)1   -5.5310  2.7714  -1.9957  0.0460  -10.9628  -0.0991    *
ns(lvef_myocarditis_mean, df = 3)2    9.2193  2.8111   3.2796  0.0010    3.7097  14.7289   **
ns(lvef_myocarditis_mean, df = 3)3   -0.7938  1.5445  -0.5139  0.6073   -3.8209   2.2333

---
Signif. codes:  0 ‘***’ 0.001 ‘**’ 0.01 ‘*’ 0.05 ‘.’ 0.1 ‘ ’ 1
```

Supplementary Figure S18: Forest plot mean LV-GLS, including Bigg et al.

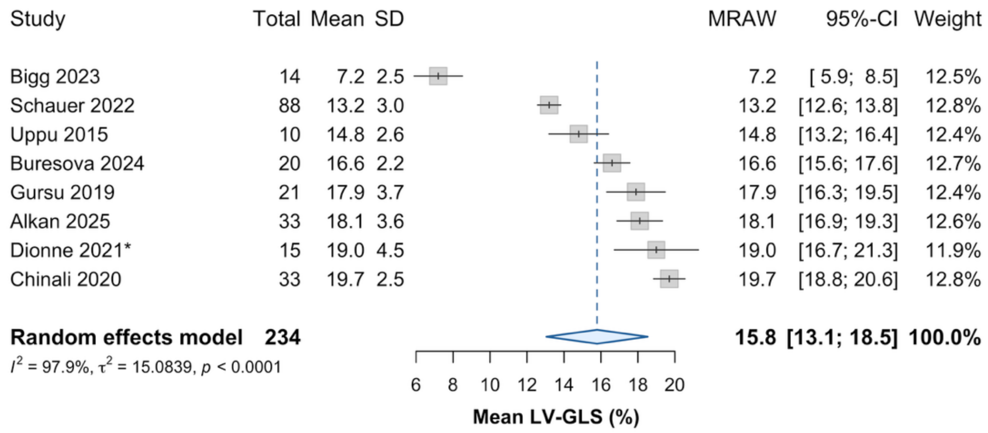

Supplementary Figure S19: Mean LV-GLS, including Bigg et al., versus age meta-regression (linear) model parameters

```
Mixed-Effects Model (k = 8; tau^2 estimator: DL)

  logLik  deviance       AIC       BIC      AICc
-20.1433   33.2068   46.2866   46.5249   52.2866

tau^2 (estimated amount of residual heterogeneity):     14.2045 (SE = 9.9348)
tau (square root of estimated tau^2 value):             3.7689
I^2 (residual heterogeneity / unaccounted variability): 97.81%
H^2 (unaccounted variability / sampling variability):   45.60
R^2 (amount of heterogeneity accounted for):            5.83%

Test for Residual Heterogeneity:
QE(df = 6) = 273.5958, p-val < .0001

Test of Moderators (coefficient 2):
QM(df = 1) = 3.9290, p-val = 0.0475

Model Results:

                      estimate      se    zval    pval   ci.lb    ci.ub
intrcpt                 9.1038  3.6358  2.5040  0.0123  1.9779  16.2298  *
age_myocarditis_mean    0.5400  0.2724  1.9822  0.0475  0.0060   1.0740  *

---
Signif. codes:  0 ‘***’ 0.001 ‘**’ 0.01 ‘*’ 0.05 ‘.’ 0.1 ‘ ’ 1
```

Supplementary Figure S20: Mean LV-GLS, including Bigg et al., versus age meta-regression (non-linear) model parameters

```
Mixed-Effects Model (k = 8; tau^2 estimator: DL)

  logLik  deviance       AIC       BIC      AICc
-16.3965   25.7132   42.7930   43.1902   72.7930

tau^2 (estimated amount of residual heterogeneity):     6.1477 (SE = 5.1113)
tau (square root of estimated tau^2 value):             2.4795
I^2 (residual heterogeneity / unaccounted variability): 94.87%
H^2 (unaccounted variability / sampling variability):   19.49
R^2 (amount of heterogeneity accounted for):            59.24%

Test for Residual Heterogeneity:
QE(df = 4) = 77.9614, p-val < .0001

Test of Moderators (coefficients 2:4):
QM(df = 3) = 14.8261, p-val = 0.0020

Model Results:

                                  estimate      se     zval    pval    ci.lb    ci.ub
intrcpt                             7.1831  2.5679   2.7973  0.0052   2.1501  12.2160  **
ns(age_myocarditis_mean, df = 3)1   7.4847  4.3685   1.7133  0.0867  -1.0775  16.0468   .
ns(age_myocarditis_mean, df = 3)2  18.1128  7.1026   2.5502  0.0108   4.1919  32.0336   *
ns(age_myocarditis_mean, df = 3)3  -0.1262  3.7929  -0.0333  0.9734  -7.5603   7.3078

---
Signif. codes:  0 ‘***’ 0.001 ‘**’ 0.01 ‘*’ 0.05 ‘.’ 0.1 ‘ ’ 1
```

Supplementary Figure S21: Mean LV-GLS versus age meta-regression (linear) model parameters

```
Mixed-Effects Model (k = 7; tau^2 estimator: DL)

  logLik  deviance       AIC       BIC      AICc
-14.5969   23.1455   35.1938   35.0315   43.1938

tau^2 (estimated amount of residual heterogeneity):     5.4538 (SE = 4.1145)
tau (square root of estimated tau^2 value):             2.3353
I^2 (residual heterogeneity / unaccounted variability): 93.98%
H^2 (unaccounted variability / sampling variability):   16.61
R^2 (amount of heterogeneity accounted for):            37.21%

Test for Residual Heterogeneity:
QE(df = 5) = 83.0468, p-val < .0001

Test of Moderators (coefficient 2):
QM(df = 1) = 1.7981, p-val = 0.1799

Model Results:

                      estimate      se     zval    pval    ci.lb    ci.ub
intrcpt                26.8146  7.3803   3.6333  0.0003  12.3496  41.2797  ***
age_myocarditis_mean   -0.6943  0.5178  -1.3409  0.1799  -1.7091   0.3205

---
Signif. codes:  0 ‘***’ 0.001 ‘**’ 0.01 ‘*’ 0.05 ‘.’ 0.1 ‘ ’ 1
```

Supplementary Figure S22: Mean LV-GLS versus age meta-regression (non-linear) model parameters

```
Mixed-Effects Model (k = 7; tau^2 estimator: DL)

  logLik  deviance       AIC       BIC      AICc
-12.4071   18.7659   34.8142   34.5438   94.8142

tau^2 (estimated amount of residual heterogeneity):     3.3272 (SE = 3.3977)
tau (square root of estimated tau^2 value):             1.8241
I^2 (residual heterogeneity / unaccounted variability): 88.85%
H^2 (unaccounted variability / sampling variability):   8.97
R^2 (amount of heterogeneity accounted for):            61.69%

Test for Residual Heterogeneity:
QE(df = 3) = 26.9027, p-val < .0001

Test of Moderators (coefficients 2:4):
QM(df = 3) = 6.3577, p-val = 0.0954

Model Results:

                                   estimate      se     zval    pval     ci.lb    ci.ub
intrcpt                             16.0656  1.7323   9.2739  <.0001   12.6703  19.4609  ***
ns(age_myocarditis_mean, df = 3)1   -3.5898  3.0210  -1.1883  0.2347   -9.5108   2.3313
ns(age_myocarditis_mean, df = 3)2    5.7693  5.7500   1.0033  0.3157   -5.5006  17.0392
ns(age_myocarditis_mean, df = 3)3   -6.7882  2.7345  -2.4825  0.0130  -12.1477  -1.4287    *

---
Signif. codes:  0 ‘***’ 0.001 ‘**’ 0.01 ‘*’ 0.05 ‘.’ 0.1 ‘ ’ 1
```

Supplementary Figure S23: Subgroup forest plot mean LV-GLS vs aetiology

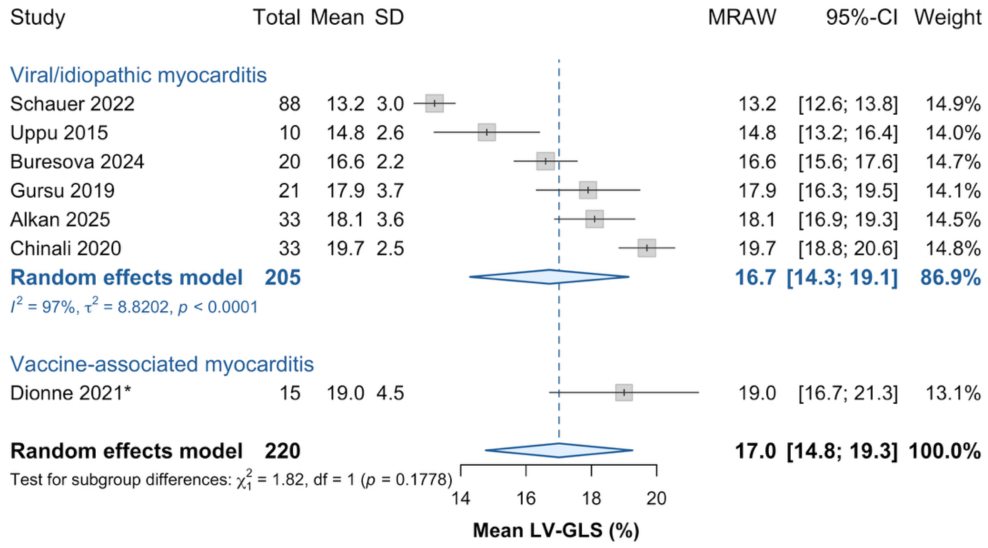

Supplementary Figure S24: Subgroup meta-analysis mean LV-GLS vs aetiology model parameters

```
                 mean             95%-CI %W(random)                      aetiology
Schauer 2022  13.2000 [12.5732; 13.8268]       14.9   Viral/idiopathic myocarditis
Uppu 2015     14.8000 [13.1885; 16.4115]       14.0   Viral/idiopathic myocarditis
Buresova 2024 16.6000 [15.6358; 17.5642]       14.7   Viral/idiopathic myocarditis
Gursu 2019    17.9000 [16.3175; 19.4825]       14.1   Viral/idiopathic myocarditis
Alkan 2025    18.1000 [16.8717; 19.3283]       14.5   Viral/idiopathic myocarditis
Dionne 2021*  19.0000 [16.7227; 21.2773]       13.1 Vaccine-associated myocarditis
Chinali 2020  19.7000 [18.8470; 20.5530]       14.8   Viral/idiopathic myocarditis

Number of studies: k = 7
Number of observations: o = 220

                        mean             95%-CI
Random effects model 17.0116 [14.7667; 19.2566]

Quantifying heterogeneity (with 95%-CIs):
 tau^2 = 8.6852 [2.4525; 35.7704]; tau = 2.9471 [1.5660; 5.9808]
 I^2 = 96.6% [94.8%; 97.8%]; H = 5.41 [4.36; 6.70]

Test of heterogeneity:
      Q d.f.  p-value
 175.55    6 < 0.0001

Results for subgroups (random effects model):
                                             k    mean             95%-CI  tau^2    tau      Q   I^2
aetiology = Viral/idiopathic myocarditis     6 16.7126 [14.2869; 19.1384] 8.8202 2.9699 169.27 97.0%
aetiology = Vaccine-associated myocarditis   1 19.0000 [16.7227; 21.2773]     --     --   0.00    --

Test for subgroup differences (random effects model):
                  Q d.f. p-value
Between groups 1.82    1  0.1778

Details of meta-analysis methods:
- Inverse variance method
- DerSimonian-Laird estimator for tau^2
- Jackson method for confidence interval of tau^2 and tau
- Calculation of I^2 based on Q
- Untransformed (raw) means
```

LV-GCS

Pooled LV-GCS

Supplementary Figure S25: Subgroup forest plot mean LV-GCS vs LVEF status

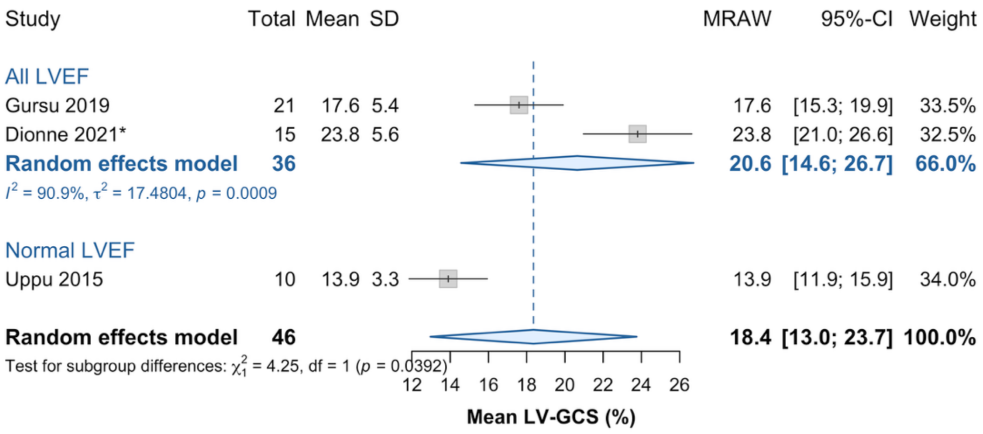

Supplementary Figure S26: Subgroup meta-analysis mean LV-GCS vs LVEF status model parameters

```
                  mean             95%-CI %W(random)    lvef_norm
Uppu 2015    13.9000 [11.8547; 15.9453]       34.0 Normal LVEF
Gursu 2019    17.6000 [15.2904; 19.9096]       33.5    All LVEF
Dionne 2021* 23.8000 [20.9661; 26.6339]       32.5    All LVEF

Number of studies: k = 3
Number of observations: o = 46

                         mean             95%-CI
Random effects model 18.3585 [12.9718; 23.7452]

Quantifying heterogeneity (with 95%-CIs):
 tau^2 = 21.1452 [4.9805; >224.1664]; tau = 4.5984 [2.2317; >14.9722]
 I^2 = 93.5% [84.4%; 97.3%]; H = 3.93 [2.54; 6.09]

Test of heterogeneity:
     Q d.f.  p-value
 30.87    2 < 0.0001

Results for subgroups (random effects model):
                          k    mean             95%-CI   tau^2    tau     Q   I^2
lvef_norm = All LVEF      2 20.6434 [14.5685; 26.7183] 17.4804 4.1810 11.05 90.9%
lvef_norm = Normal LVEF   1 13.9000 [11.8547; 15.9453]      --     --  0.00    --

Test for subgroup differences (random effects model):
                  Q d.f. p-value
Between groups 4.25    1  0.0392

Details of meta-analysis methods:
- Inverse variance method
- DerSimonian-Laird estimator for tau^2
- Jackson method for confidence interval of tau^2 and tau
- Calculation of I^2 based on Q
- Untransformed (raw) means
```

Supplementary Figure S27: Subgroup forest plot mean LV-GCS vs aetiology

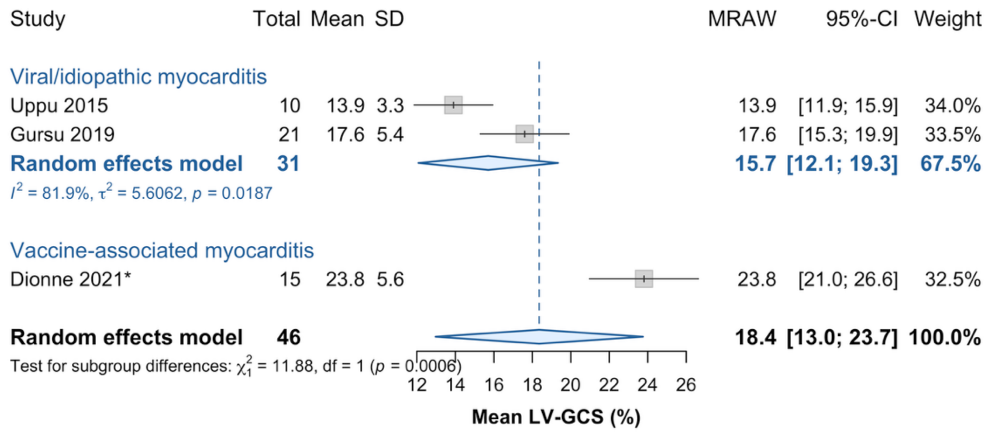

Supplementary Figure S28: Subgroup meta-analysis mean LV-GCS vs aetiology model parameters

```
                mean            95%-CI %W(random)                   aetiology
Uppu 2015    13.9000 [11.8547; 15.9453]       34.0   Viral/idiopathic myocarditis
Gursu 2019   17.6000 [15.2904; 19.9096]       33.5   Viral/idiopathic myocarditis
Dionne 2021* 23.8000 [20.9661; 26.6339]       32.5 Vaccine-associated myocarditis

Number of studies: k = 3
Number of observations: o = 46

                        mean            95%-CI
Random effects model 18.3585 [12.9718; 23.7452]

Quantifying heterogeneity (with 95%-CIs):
 tau^2 = 21.1452 [4.9805; >224.1664]; tau = 4.5984 [2.2317; >14.9722]
 I^2 = 93.5% [84.4%; 97.3%]; H = 3.93 [2.54; 6.09]

Test of heterogeneity:
     Q d.f.  p-value
 30.87    2 < 0.0001

Results for subgroups (random effects model):
                                             k    mean             95%-CI  tau^2    tau    Q   I^2
aetiology = Viral/idiopathic myocarditis     2 15.7095 [12.0845; 19.3346] 5.6062 2.3677 5.53 81.9%
aetiology = Vaccine-associated myocarditis   1 23.8000 [20.9661; 26.6339]     --     -- 0.00    --

Test for subgroup differences (random effects model):
                   Q d.f. p-value
Between groups 11.88    1  0.0006

Details of meta-analysis methods:
- Inverse variance method
- DerSimonian-Laird estimator for tau^2
- Jackson method for confidence interval of tau^2 and tau
- Calculation of I^2 based on Q
- Untransformed (raw) means
```

Supplementary Figure S29: Mean LV-GCS, versus age meta-regression (linear) model parameters

```
Mixed-Effects Model (k = 3; tau^2 estimator: DL)

  logLik  deviance       AIC       BIC      AICc
 -8.9498   11.2349   23.8996   21.1954   47.8996

tau^2 (estimated amount of residual heterogeneity):     42.9715 (SE = 63.3124)
tau (square root of estimated tau^2 value):             6.5553
I^2 (residual heterogeneity / unaccounted variability): 95.99%
H^2 (unaccounted variability / sampling variability):   24.91
R^2 (amount of heterogeneity accounted for):            0.00%

Test for Residual Heterogeneity:
QE(df = 1) = 24.9117, p-val < .0001

Test of Moderators (coefficient 2):
QM(df = 1) = 0.1211, p-val = 0.7278

Model Results:

                      estimate       se     zval    pval     ci.lb    ci.ub
intrcpt                28.4555  29.1620   0.9758  0.3292  -28.7009  85.6120
age_myocarditis_mean   -0.6811   1.9569  -0.3480  0.7278   -4.5166   3.1545

---
Signif. codes:  0 ‘***’ 0.001 ‘**’ 0.01 ‘*’ 0.05 ‘.’ 0.1 ‘ ’ 1
```

LVEDD

Pooled LVEDD

Supplementary Figure S30: Forest plot mean LVEDD

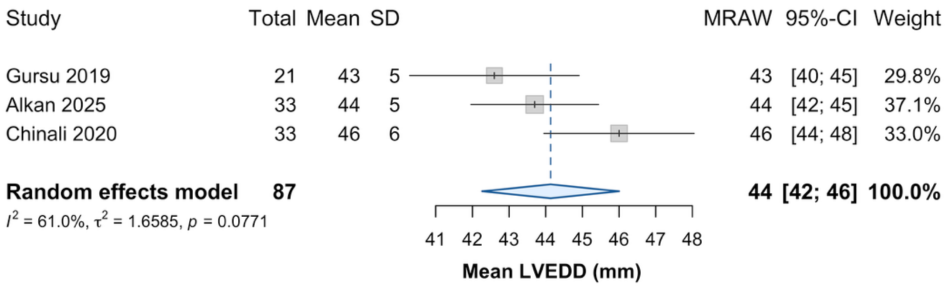

Supplementary Figure S31: Mean LVEDD versus age meta-regression (linear) model parameters

```
Mixed-Effects Model (k = 3; tau^2 estimator: DL)

  logLik  deviance       AIC       BIC      AICc
 -5.9048    6.1187   17.8096   15.1054   41.8096

tau^2 (estimated amount of residual heterogeneity):     4.7593 (SE = 8.4507)
tau (square root of estimated tau^2 value):             2.1816
I^2 (residual heterogeneity / unaccounted variability): 79.65%
H^2 (unaccounted variability / sampling variability):   4.91
R^2 (amount of heterogeneity accounted for):            0.00%

Test for Residual Heterogeneity:
QE(df = 1) = 4.9133, p-val = 0.0267

Test of Moderators (coefficient 2):
QM(df = 1) = 0.0117, p-val = 0.9137

Model Results:

                      estimate       se     zval    pval     ci.lb    ci.ub
intrcpt                45.9725  17.1806   2.6758  0.0075   12.2992  79.6458  **
age_myocarditis_mean   -0.1401   1.2932  -0.1084  0.9137   -2.6747   2.3944

---
Signif. codes:  0 ‘***’ 0.001 ‘**’ 0.01 ‘*’ 0.05 ‘.’ 0.1 ‘ ’ 1
```
